# PM2.5-Related Neonatal Infections: A Global Burden Study from 1990 to 2019

**DOI:** 10.3390/ijerph19095399

**Published:** 2022-04-28

**Authors:** Zeyu Tang, Jinzhu Jia

**Affiliations:** 1 Department of Biostatistics, School of Public Health, Peking University, No.38, Xueyuan Road, Beijing 100871, China; tangzeyu@pku.edu.cn; 2 Center for Statistical Science, Peking Universeity, 5 Summer Palace Road, Beijing 100871, China

**Keywords:** fine particulate matter, neonates, environment, air pollution, socio-demographic index

## Abstract

Background: Long-term exposure to fine particulate matter (PM2.5) may increase the risk of neonatal infections. To show the effects of PM2.5 on neonatal infections as well as the trends of the effect, we studied the burden measured by the age-standardized mortality rate (ASMR) and the age-standardized disability-adjusted life years rate (ASDR) and its trends with the socio-demographic index in 192 countries and regions from 1990 to 2019. Methods: This is a retrospective study that uses the Global Burden of Disease Study 2019 database. The age-standardized mortality rate and age-standardized disability-adjusted life years rate are used to measure the burden of PM2.5-related neonatal infections in different countries and regions. The annual percentage changes and the average annual percentage changes are used to reflect the trends over the years (1990–2019) and are calculated using a Joinpoint model. The relationship of the socio-demographic index with the ASMR and ASDR is calculated and described using Gaussian process regression. Results: With the rapid increase in the global annual average of PM2.5, the global burden of PM2.5-related neonatal infections has increased since 1990, especially in early neonates, boys, and low-middle SDI regions. Globally, the ASMR and ASDR of PM2.5-related neonatal infections in 2019 were 0.21 (95% CI: 0.14, 0.31) and 19.06 (95% CI: 12.58, 27.52) per 100,000 people, respectively. From 1990 to 2019, the ASMR and ASDR increased by 72.58% and 73.30%, and their average annual percentage changes were 1.9 (95% CI: 1.3, 2.6) and 2.0 (95% CI: 1.3, 2.6), respectively. When the socio-demographic index was more than 0.60, it was negatively related to the burden of PM2.5-related neonatal infections. Surprisingly, the burden in low SDI regions was lower than it was in low-middle and middle SDI regions, while high-middle and high-SDI regions showed decreasing trends. Interpretation: Boys bore a higher PM2.5-related neonatal burden, with male fetuses being more likely to be affected by prenatal exposure to PM2.5 and having less of a biological survival advantage. Poverty was the root cause of the burden. Higher SDI countries devoted more resources to improving air quality, the coverage of medical services, the accessibility of institutional delivery, and timely referral to reduce the disease burden. The burden in low SDI regions was lower than that in low-middle and middle SDI regions. One reason was that the benefits of medical services were lower than the harm to health caused by environmental pollution in low-middle and middle SDI regions. Moreover, the underreporting of data is more serious in low SDI countries. Conclusions: In the past 30 years, the global burden of PM2.5-related neonatal infections has increased, especially in early neonates, boys, and low-middle SDI regions. The huge difference compared to higher SDI countries means that lower SDI countries have a long way to go to reduce the disease burden. Policy makers should appropriately allocate medical resources to boys and early newborns and pay more attention to data under-reporting in low SDI countries. In addition, it is very necessary to promulgate policies to prevent and control air pollution in countries with large and increasing exposure to PM2.5 pollution.

## 1. Introduction

There are 130 million babies born every year, approximately 4 million of which die during the neonatal period—the first 4 weeks of life [1]. Followed by complications from preterm birth and intrapartum-related conditions, infections are the third leading cause of neonatal deaths [2]. In 2012, there were 0.66 million newborns who died of infections, accounting for 23% of all neonatal deaths [2]. In addition, 48% of newborns died in the late neonatal period due to infections, comprising the main cause of death during this period [2].

PM2.5 is associated with neonatal infections and other adverse neonatal outcomes, including a low birth weight and being small for the baby’s gestational age [3,4,5]. Asim Anwar et al. found that PM2.5 is positively related to neonatal deaths that can be attributed to acute respiratory infection [6]. Influenza virus infection in infants becomes more serious when the infant has increased exposure to particulate matter [7]. For children who are under two years old, an increase in the 30-day average PM2.5 exposure to 25 µg/m^3^ was associated with a 1.29-fold elevation in the daily rate of bronchitis [8]. There are some PM2.5 mechanisms that lead to neonatal infections. Infection is one of the main causes of early neonatal death, in which immunity plays an important role [1,9]. Immune system development is essential for health, especially in the prenatal period. PM2.5 affects the development of the immune system, and there are some studies supporting the impact of early exposure on the immune system [10,11,12]. It was found that exposure to PM2.5 during pregnancy changes the distribution of lymphocytes the in umbilical cord blood, something that was later reflected the immune ability of newborns [13]. Studies have found that increasing the number of T lymphocytes and decreasing the number of B lymphocytes and of natural killer cells were related to PM2.5 exposure during early pregnancy, while a decrease in the number of T lymphocytes and an increase in the number of B lymphocytes and natural killer cells were related to PM2.5 exposure during late pregnancy [14].

Globally, the distribution of PM2.5 is uneven both temporally and spatially. From 1998 to 2014 and mainly driven by Asian and African regions, the population-weighted annual average PM2.5 concentration globally were three times more than those recommended by the World Health Organization [15]. The change in the annual PM2.5 concentrations showed positive trends in India (1.13 ± 0.15 μg/m^3^/year) and globally (0.04 ± 0.02 μg/m^3^/year) and negative trends in eastern North America (−0.28 ± 0.03 μg/m^3^/year) and Europe (−0.15 ± 0.03 μg/m^3^/year) from 1998 to 2018.

The socio-demographic index is a newly established classification index that was used in the Global Burden of Disease Study in 2015. It is calculated by income per capita, educational attainment, and fertility [16]. This index is scaled. More and more studies have used the socio-demographic index to assess disease-related burden in several countries [17,18,19]. For example, Liu et.al studied the global burden of type 2 diabetes attributable to PM2.5. They analyzed the trends over the past 30 years. However, little is known about the burden of PM2.5-related neonatal infections in different countries or regions. Additionally, the global trends between the socio-demographic index and the burden in the last 30 years are also unknown. In this study, we summarized the burden of PM2.5-related neonatal infections in different countries and regions for the last 30 years and determined the differences in different age and sex groups. We modeled the relationship between PM2.5-related neonatal infection burden and time in 192 countries and regions and analyzed the change trends among them. We also established a model to analyze the trends of infection burden with socio-demographic indexes, analyzed the possible reasons for these trends, and provided reasonable suggestions to formulate relevant policies.

## 2. Materials and Methods

### 2.1. Study Data

We obtained summarized data on PM2.5-related neonatal infections covering the period from 1990 to 2019 from the Global Burden of Disease Study 2019 (GBD 2019) using the Global Health Data Exchange (GHDx, http://ghdx.healthdata.org/gbd-results-tool, accessed on 4 January 2022) [20,21,22]. The GBD 2019 data are contained in a well-designed dataset, and missing data have been properly handled [20]. Some high-level studies have been conducted on the GBD 2019 data [19,21,22]. The data contain PM2.5 values, age-standardized mortality rates (ASMR, per 100,000 people), age-standardized disability-adjusted life year rates (ASDR, per 100,000 people), causes, risk factors, and socio-demographic indexes for 192 countries and regions around the world from 1990 to 2019.

The age-standardized mortality rate is defined as a weighted average of the age-specific mortality rates per 100,000 people, and the weights are the proportion of people in the corresponding WHO age groups for the standard population. The age-standardized disability-adjusted life year rate is disability-adjusted life years, which are adjusted for differences in the age distribution of the population and then expressed per 100,000 people (www.who.int/data/gho/indicator-metadata-registry, accessed on 4 January 2022). Disability-adjusted life years is the sum of the years of life lost because of premature mortality and the number of years lived with a disability because of the prevalence of a disease or health condition in a population (www.who.int/data/gho/indicator-metadata-registry, accessed on 4 January 2022).The years of life lost because of premature mortality are the numbers of deaths and lives lost due to early death, while the years lived with a disability are any healthy life years lost in the short or long term as a result of disability and are weighted by disability weight [22].

According to the International Classification of Diseases, Tenth Revision (ICD-10), neonatal sepsis and other neonatal infections in the Global Burden of Disease Study 2017 were defined by the codes P36–P36.9, P38–P39.9. Satellite-based estimates, chemical transport model simulations, and ground measurements were used to calculate the annual average PM2.5 at an approximate 11 km × 11 km resolution. Then, it was used to determine the population-weighted mean concentrations [23,24]. The socio-demographic index is used to reflect the level of social development and is scaled between 0 and 1. If a region or country has an index score of 1.0, it means that this region or country has the highest observed level of educational attainment, the highest log income per capita, and the lowest fertility rate [16]. The lower the index, the lower the social development level of the region. There are five socio-demographic index (SDI) regions: high SDI regions (>0.81), high-middle SDI regions (0.70–0.81), middle SDI regions (0.61–0.69), low-middle SDI regions (0.46–0.60), and low SDI regions (<0.46) [22].

### 2.2. Statistical Analysis

The age-standardized mortality rate (ASMR)and age-standardized disability-adjusted life years rate (ASDR)were used to evaluate the burden of PM2.5-related neonatal infections because age structures are different among countries and regions. We calculated the percentage changes, annual percentage changes (APCs), and average annual percentage changes (AAPCs) using a Joinpoint model and set two knots for each region or country. The APCs and AAPCs reflected the trends in the ASMR and ASDR related to PM2.5 between 1990 and 2019 [22]. The APCs were used to measure the linear change trend of each section, and AAPCs are used to measure the global change trends. The APCs and AAPCs were calculated as follows:

(1) $$\ln\left(\mathrm{ASMR}\;\mathrm{or}\;\mathrm{ASDR}\right)=\alpha +\beta_{i}x,\;\mathrm{APCs}=100\times \left(\exp\left(\beta_{i}\right)-1\right),\;\mathrm{AAPCs}=\left\{\exp\left(\sum w_{i}\beta_{i}/\sum w_{i}\right)-1\right\}\times 10$$

where x indicates the year, $\beta_{i}$ is the slope coefficient in each section spilt by knots, and $w_{i}$ is the range of the year in each section.

To access the trends between the ASMR, ASDR, and socio-demographic indexes (SDI), we calculated the Pearson correlation coefficients when the SDI was <0.44, 0.44–0.60, and >0.60, respectively. The Gaussian process regression model was used to calculate the expected value [25]. It has previously been used to analyze trends in other studies [22,26].

Gaussian process regression can be used to model functional data, and it captures the correlation structure between outcomes. These correlation structures may depend on explanatory variables. Here, we assumed that disease burden is related to socio-demographic indexes. If two countries have similar graphic indexes, then their disease burdens have a strong correlation. This observation can be expressed as

(2) $$y_{i}=t\left(x_{i}\right)+\varepsilon_{i}$$

where $y_{i}$ is the burden of PM2.5-related neonatal infections, $x_{i}$ is the SDI index, $\varepsilon_{i}$ is a Gaussian distribution with a mean of 0, and its covariance matrix is specified by a kernel matrix K.

We used R software [27] to conduct all of the calculations and to draw all pictures, with the exception off the Joinpoint model and its related pictures [28]. The “kernlab” package in R was used to build Gaussian process regression model with gausspr = “rbfdot” [29]. Details about Gaussian process regression and how to perform it in R are available at Vignettes of the “kernlab” package [29]). The “maps” package and “ggplot2” in R were used to draw pictures [30,31]. Joinpoint software was used to build the Joinpoint model and to draw any related pictures [32].

## 3. Results

### 3.1. Global PM2.5-Related Neonatal Infections Burden from 1990 to 2019

**Figure 1:**
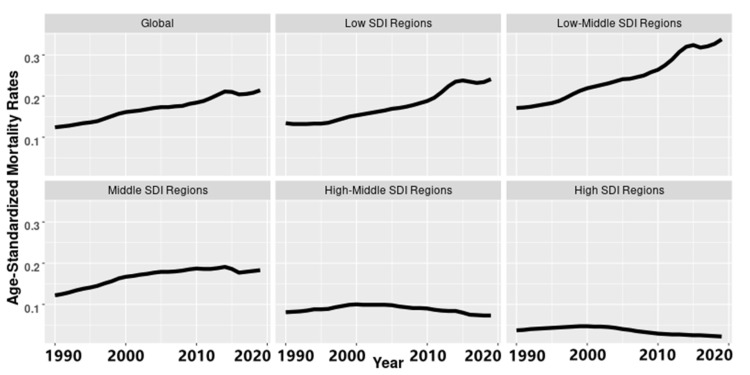
The changes in the age-standardized mortality rate in PM2.5-related neonatal infections according to the global population and various socio-demographic index (SDI) regions from 1990 to 2019.

We analyzed the trends of the age-standardized mortality rate (ASMR) and age-standardized disability-adjusted life years rate (ASDR) for PM2.5-related neonatal infections in 192 countries and regions from 1990 to 2019. The burden was still high in 2019, especially in early neonates, boys, and in low-middle SDI regions. The ASMR and ASDR of the PM2.5-related neonatal infections were 0.21 and 19.06 per 100 thousand people in 2019, respectively (Table 1 and Table 2). PM2.5-related neonatal infections have increased globally since 1990, especially in low, low-middle, and middle SDI regions (Figure 1 and Figure S43).

The highest ASMR and ASDR in PM2.5-related neonatal infections were found in Western Sub-Saharan Africa (0.49 and 43.40 per 100 thousand people) followed by Southern Sub-Saharan Africa (0.36 and 31.86 per 100 thousand people) and South Asia (0.35 and 31.04 per 100 thousand people); however, Australasia had the lowest ASMR and ASDR (0.006 and 0.50 per 100 thousand people). Globally, the ASMR and ASDR increased by 72.58% and 73.30%, respectively, from 1990 to 2019. The Caribbean had the largest increase in the ASMR and ASDR (percentage change: 111.34% and 110.80%), while Central Europe had the largest decline (percentage change: −68.09% and −69.44%).

From 1990 to 2019, the burden of PM2.5-related neonatal infections decreased in 7 of 21 regions, with the average annual percentage changes of in the ASMR ranging from −1.7 (95% CI: −2.2, −1.2) in Eastern Europe to −4.1 (95% CI: −4.6, −3.5) in Central Europe. In contrast, 9 of 21 regions showed an increasing trend, where the changes ranged from 1.4 (95% CI: 1, 1.8) in Oceania to 2.7 (95% CI: 2.3, 3.1) in the Caribbean. The average annual percentage changes in the ASDR showed a similar trend, of which the minimum values were −4.2 (95% CI: −4.6, −3.7) in Central Europe, and the maximum value was 2.7 (95% CI: 2.3, 3.1) in the Caribbean (Table 1 and Table 2). As seen in Table 1 and Table 2, there were three-stage annual percentage changes in the ASMR and ASDR in 21 regions from 1990 to 2019. More details are shown in the Supplementary Materials (Figures S1–S42).

### 3.2. PM2.5-Related Neonatal Infections Burden in Different SDI Regions

Compared to other regions, the low-middle SDI regions had the highest age-standardized mortality rate in 2019, 0.34 (95% CI: 0.22, 0.48), as well as the largest increase in it from 1990 to 2019, demonstrating an increase of 97.66% (Table 1). In contrast, high SDI regions enjoyed not only the lowest age-standardized mortality rate, 0.022 (95% CI: 0.016, 0.030), but also the greatest decrease in it, by 40.54% (Table 1). From 1990 to 2019, the low, low-middle, and middle SDI regions had significant increases in the number of deaths caused by PM2.5-related neonatal infections, with the lower confidence limits of the 95% confidence intervals of the average annual percentage changes being positive (Table 1 and Table 2). High-middle and high-SDI regions had decreasing trends, where average annual percentage changes in the ASMR and ASDR ranged from −0.4 to −1.8, and −0.4 to −1.7, respectively.

The highest and the lowest ASDRs in 2019 were found in low-middle SDI and high SDI regions, respectively (Table 2). The largest decrease in the ASDR percentage change occurred in high SDI regions starting in 1990, with a change of 39.33%. In contrast, the maximum increase in the value was found in low-middle SDI regions, with an increase of 97.87% (Table 2).

**Figure 2:**
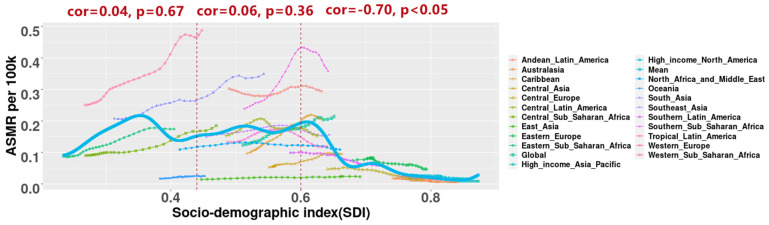
Age-standardized mortality rates (ASMR) of PM2.5-related neonatal infections globally and in 21 regions in 1990–2019. The expected value is represented by the solid blue line. The ASMR of PM2.5-related neonatal infections in four regions (South Asia, Southern Sub-Saharan Africa, Western Sub-Saharan Africa, and Andean Latin America) were obviously higher than the expected value.

When the socio-demographic index was <0.44 and 0.44–0.60, it was not associated with ASMR and ASDR. However, when it was more than 0.60, a strongly negative correlation was found among them (Figure 2 and Supplemental Figure S44). As seen from the results of the Gaussian process, the ASMR of PM2.5-related neonatal infections in South Asia, Southern Sub-Saharan Africa, Western Sub-Saharan Africa, and Andean Latin America were obviously higher than the expected value (Figure 2). However, it was lower than or similar to the expected value in other regions. The estimated relationship between the ASDR and the socio-demographic index in PM2.5-related neonatal infections was very similar to that between ASMR and the socio-demographic index (Supplemental Figure S44).

### 3.3. PM2.5-Related Neonatal Infections Burden in Different Age Groups and Genders

**Figure 3:**
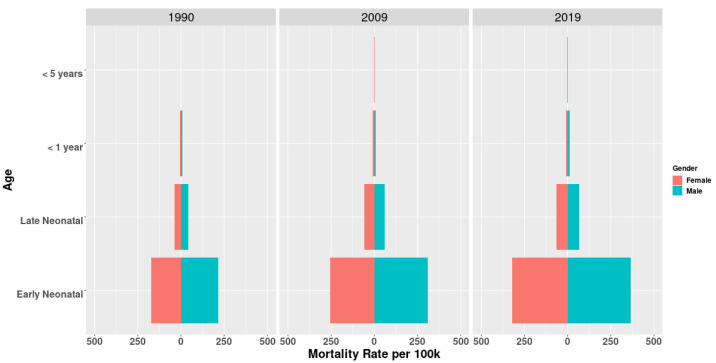
Global mortality rates (per 100 thousand) in different age groups and genders for PM2.5-related neonatal infections in 1990, 2009 and 2019.

**Figure 4:**
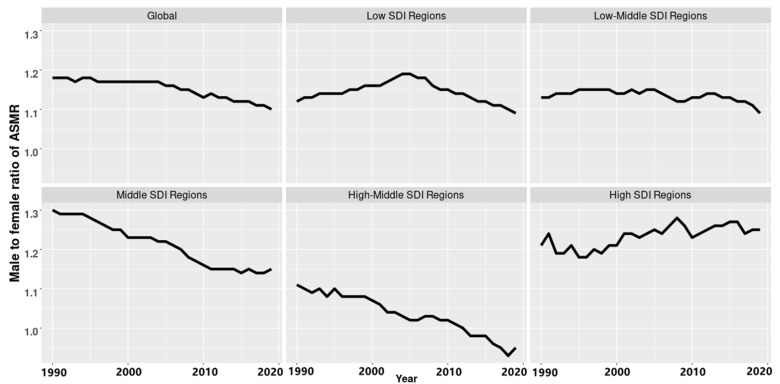
The ratio of males to females in age-standardized mortality rates (ASMR) for PM2.5-related neonatal infections in different socio-demographic index (SDI) regions in 1990–2019.

Similar results can be seen in different age groups and in different socio-demographic index groups. The ASMR of PM2.5-related neonatal infections was obviously higher in males than it was in females (Figure 3 and Figure 4). Deaths due to PM2.5-related neonatal infections were almost concentrated in early neonates followed by late neonates. High SDI regions showed an increasing trend in the sex ratio of the ASMR starting in 1990, while middle and high-middle SDI regions showed a significant decline and remained stable in other regions. Such trends in the ASDR were similar to those observed in the ASMR (Supplemental Figure S45).

### 3.4. PM2.5-Related Neonatal Infections Burden by Countries

**Figure 5:**
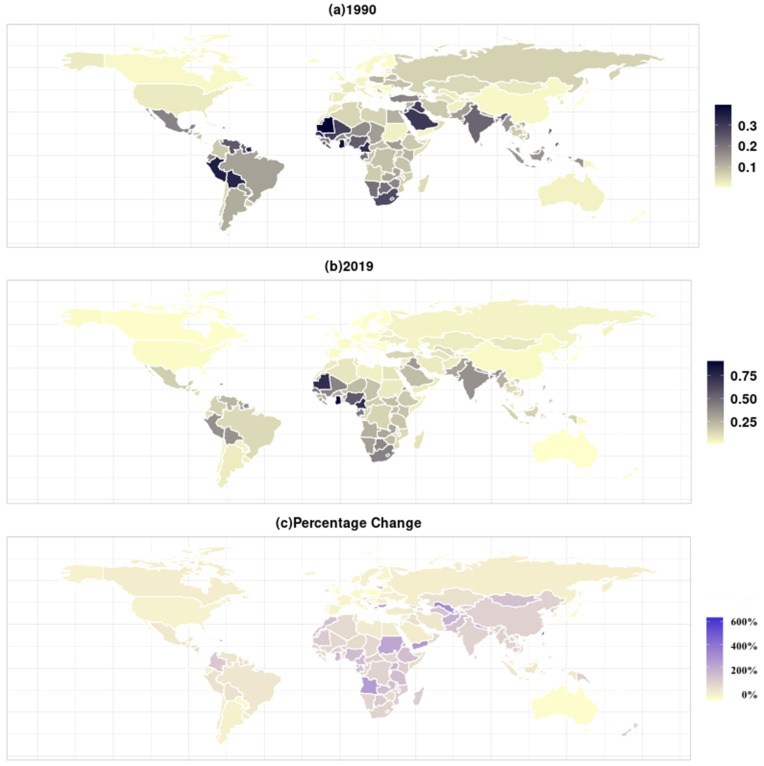
Age-standardized mortality rates (per 100 thousand) for PM2.5-related neonatal infections globally in (**a**) 1990 and (**b**) 2019 and (**c**) percentage changes in 1990–2019.

Globally, the burden of PM2.5-related neonatal infections varied widely among different countries in 1990 and 2019 (Figure 5). In 2019, the age-standardized mortality rate (ASMR) 300-fold variation, ranging from 0.003 (per 100 thousand population) in four countries (Albania, Cyprus, Greece, Luxembourg) to 0.907 (per 100 thousand people) in Ghana. It was more than 0.5 per 100 thousand people in six countries (Cameroon, Djibouti, Ghana, Equatorial Guinea, Mauritania, Nigeria) and under 0.3 per 100 thousand people in 168 countries. As shown in Table 1 and Table 2, the burden PM2.5-related neonatal infections in five regions (Caribbean, Andean Latin America, South Asia, Western Sub-Saharan Africa, Southern Sub-Saharan Africa) were higher than it was in other regions. Conversely, six regions (Australasia, Western Europe, High-income Asia Pacific, Oceania, East Asia, High-income North America) had a relatively low PM2.5-related neonatal infection burden.

Between 1990 and 2019, the ASMR of the PM2.5-related neonatal infections declined most in 22 countries, decreasing by more than 50%. On the other hand, Equatorial Guinea, North Macedonia, Taiwan (Province of China), and Uzbekistan had the highest increase rates, more than 300% (Figure 5). In 1990–2019, there were seven regions with a lower PM2.5-related neonatal infection burden (Central Europe, Australasia, Eastern Europe, Western Europe, Southern Latin America, High-income Asia Pacific, High income North America), while the regions with increased burden were Central Asia, Caribbean, Oceania, East Asia, South Asia, Central, Eastern, Southern, and Western Sub-Saharan Africa.

## 4. Discussion

We summarized the PM2.5-related neonatal infections burden measured according to the age-standardized mortality rate (ASMR) and age-standardized disability-adjusted life years rate (ASDR). We modeled the relationship between the PM2.5-related neonatal infection burden and time in 192 countries and regions and analyzed the change trends among them. We also established a Gaussian process regression model to analyze the trends of the burden with the socio-demographic index (SDI). With the global annual average of PM2.5 increasing rapidly, the global burden of PM2.5-related neonatal infections has increased since 1990, especially in early neonates, boys, and low-middle SDI regions. When the socio-demographic index was more than 0.60, it was negatively associated with the burden. Surprisingly, the burden in low SDI regions was lower than that in low-middle and middle SDI regions, while high-middle and high-SDI regions showed decreasing trends.

The burden of PM2.5-related neonatal infections varied greatly among different socio-demographic index regions, with the ASMR ranging from 0.022 in high SDI regions to 0.34 in low-middle regions in 2019 (Table 1). When the socio-demographic index was <0.44 and 0.44–0.60, we did not find that it was significantly associated with PM2.5-related neonatal infection burden. However, when more than 0.60, there was a strongly negative relationship among them. Overall, the PM2.5-related neonatal burden was higher in lower SDI regions than it was in higher SDI regions. Among different levels of socio-demographic index regions, the difference in the PM2.5-related neonatal infection burden reflects social inequalities in the treatment of neonatal infections, health care, and air pollution control.

The trend for ambient particulate matter pollution increased due to industrialization and then decreased due to the management of air-quality in countries with higher levels of SDI [33,34]. Globally, middle and low-middle SDI regions contributed greatly to the exposure to ambient particulate matter pollution. Moreover, an estimated 4 million neonatal deaths occurred worldwide in 2000, only 1% of which occurred in the world’s 39 high-income countries, and the remaining 99% occurring in low-income and middle-income countries [35]. To some extent, these figures reflect the inequality among countries with different socio-demographic indexes.

There are several possible reasons why the PM2.5-related neonatal infection burden is lower in regions with a higher SDI than it is in regions with a lower SDI. First, the societies in high-SDI countries are more likely focusing on improving their levels of health and environment protection [36], thus contributing to reducing PM2.5 pollution levels [24]. However, the level of exposure to PM2.5, especially household air pollution exposure, is still higher in lower SDI regions than it is in higher SDI regions [20]. Second, poverty plays a crucial role in causing neonatal deaths by impeding access to health care or increasing the prevalence of related risk factors, such as maternal infection [1]. A disparity exists in neonatal deaths and in other adverse outcomes between the richest and poorest 20% of Canadians [37]. Based on DHS data from 20 countries in sub-Saharan Africa and three large countries in south Asia, neonatal mortality rates were higher in the poorest 20% of households than in the top quintile [1]. Third, the differences in health-care coverage, according to information regarding policy and programs, between higher and lower SDI regions are important reasons for the differences in the burden of PM2.5-related neonatal infections. Globally, the percentage of women delivering their children with a skilled attendant varied from 5% to 99% [38]. However, the rates of skilled attendants and institutional delivery are low in poor countries [1]. For instance, less than 40% of women in sub-Saharan Africa could deliver with skilled care, and the figure is less than 30% in south Asia. Early neonatal deaths account for 75% of all neonatal deaths, and preventing these depends on drawing attention to the causes of death that are unique to the first week of life. About 14% of women can access skilled care when giving birth in the countries with the highest mortality; however, that figure accounts for 86% of the richest 20% of women in these countries. Increasing the coverage of care during childbirth and during the early neonatal period to the poorest populations can help to lower neonatal mortality rates in such settings [1]. Forth, it is essential for ill babies to have access to referrals on time. A Ugandan study suggested that only 21% of severely ill babies could complete their referral as advised [39]. A lack of money is the most common reason for not completing a referral (90%) [1].

Nevertheless, we noted that the burden of PM2.5-related neonatal infections in low SDI regions is lower than that in low-middle SDI regions (Table 1 and Table 2). The possible reason is this is because the benefits of maternal and neonatal health care and facility-based delivery are lower than the harm to health caused by environmental pollution in low-middle and middle SDI regions. Another possible reason is the under-reporting of neonatal infection data in low SDI countries. In poor communities, many unnamed babies died, and their deaths were unrecorded. This could mean that the burden is underestimated in low SDI countries [1].

In high SDI countries, populations with better access to institutional delivery, skilled care, and timely referral for severely ill babies as well as continuously improving air quality have resulted in the PM2.5-related neonatal infection burden having a decreasing trend. This huge difference highlights the long way that we have to go to improve air quality and to prevent neonatal infections, particularly in countries with lower SDI levels.

We found that deaths due to PM2.5-related neonatal infections mainly occurred in early neonates, and mortality rates were lower in girls than that in boys, especially in early neonates (Figure 3 and Figure 4). There are some possible reasons to explain this phenomenon. Wieslaw Jedrychowski et.al. found that prenatal exposure to PM2.5 in the second trimester was associated with a reduction in fetal growth and that male fetuses were more susceptible than female fetuses [40]. There are some underlying explanations for this. First, male fetuses grow faster and need more oxygen than female fetuses and thus are more easily affected by carbon monoxide (CO), which plays a mediating role in the effect of air pollution on fetal growth [41,42,43]. Second, air pollution may also affect hematologic changes [44,45]. It was speculated that increased blood viscosity had more effects on male fetuses [46,47]. Third, during the late stages of pregnancy, male fetuses more easily suffered from intrauterine infections, accounting for one of the reasons why their immune systems developed slowly, and thus their amniotic membranes were more likely to become infected [48,49].

Rakesh Ghosh et al. found that males were more likely to be preterm births [50]. Preterm birth is not only a direct cause of death, but it also acts as an indirect cause of death by causing infection [51]. Moreover, preterm birth leads to long-term neurodevelopmental outcomes, such as cognitive or learning difficulties or developmental delays, cerebral palsy, epilepsy, gross motor and coordination problems, and moderate cognitive or learning difficulties [52]. Chen et al. pointed out that prenatal exposure to PM2.5 was related to neurobehavioral development in the fetus [53]. Male fetuses were also more easily affected than female fetuses.

Girls have a biological survival advantage during the neonatal period [54]. Boys are more vulnerable when facing adverse conditions, such as older maternal age and preterm birth [55,56,57]. One possible reason for this is that “endogenous” factors play an important role, such as genetic factors linked to the pseudo-dominant effect of X-linked genes [58]. Reduced care seeking for girls compared to boys has also been reported, especially in South Asia [59,60]. Although female infanticide has been reported in rural China and in South Asia, the true incidence of this practice is unknown [61,62,63]. The biological advantage of girls has a greater impact on their numbers, which may explain why the mortality rate is lower in girls than in boys.

The present study has several limitations. First, due to the lack of air monitoring stations, the PM2.5 in low-income countries may not be accurate enough, but the Global Burden of Disease data is well designed on the whole. Second, we did not assess the impact of different particulate matter sources because the data on this are limited. Third, we did not consider the differences in neonatal infections based on occupation, race, and other factors at the same time due to a lack of data.

## 5. Conclusions

In the past 30 years, the global PM2.5-related neonatal infection burden has increased, especially in low-middle SDI regions. The huge difference in higher SDI countries means that lower SDI countries have a long way to go to reduce the disease burden. Policy makers should appropriately allocate medical resources to boys and early newborns and should pay more attention to the underreporting of data in low SDI countries. In addition, it is very necessary to promulgate policies to prevent and control air pollution in countries with large and increasing amounts of exposure to PM2.5 pollution.

## Figures and Tables

**Table 1:** Age-standardized mortality rate in 1990 and 2019: changes in percentage, annual percentage changes, and average annual percentage changes in PM2.5-related neonatal infections from 1990 to 2019 globally and in different regions.

Location	ASMR in 1990	ASMR in 2019	Changes in Percentage 1990–2019	APCs (Stage1)	APCs (Stage2)	APCs (Stage3)	AAPCs from 1990 to 2019
Global	0.124 (0.065, 0.219)	0.214 (0.142, 0.31)	72.58%	2 (0.9, 3) *	4.1 (−1.9, 10.5)	1.6 (1.4, 1.8) *	1.9 (1.3, 2.6) *
Low SDI	0.134 (0.041, 0.333)	0.241 (0.122, 0.436)	79.85%	1.9 (1.8, 2.1) *	6.3 (2.8, 9.8) *	0.1 (−1.4, 1.5)	2.2 (1.7, 2.7) *
Low-middle SDI	0.171 (0.066, 0.354)	0.338 (0.216, 0.484)	97.66%	2.4 (2.2, 2.5) *	5.1 (−0.9, 11.5)	0.8 (−0.6, 2.1)	2.4 (1.7, 3) *
Middle SDI	0.122 (0.078, 0.174)	0.183 (0.139, 0.235)	50%	3.2 (2.9, 3.4) *	0.9 (0.6, 1.2) *	−0.7 (−1.2, −0.2) *	1.4 (1.2, 1.5) *
High-middle SDI	0.081 (0.06, 0.106)	0.073 (0.057, 0.092)	−9.88%	2.2 (1.9, 2.5) *	−1.3 (−1.8, −0.8) *	−2.5 (−2.9, −2.1) *	−0.4 (−0.6, −0.2) *
High SDI	0.037 (0.027, 0.051)	0.022 (0.016, 0.03)	−40.54%	2 (1.7, 2.3) *	−6.2 (−6.9, −5.5) *	−2.8 (−3.3, −2.4) *	−1.8 (−2.1, −1.6) *
Central Europe	0.047 (0.031, 0.065)	0.015 (0.01, 0.02)	−68.09%	−0.7 (−1.3, 0) *	−7.7 (−8.2, −7.2) *	−0.1 (−3.2, 3)	−4.1 (−4.6, −3.5) *
Australasia	0.018 (0.001, 0.048)	0.006 (0.001, 0.012)	−66.67%	−2.2 (−5.9, 1.5)	−10.6 (−24.4, 5.8)	−3.4 (−3.8, −3) *	−4 (−5.7, −2.2) *
Central Asia	0.053 (0.028, 0.086)	0.095 (0.057, 0.141)	79.25%	1.4 (1.2, 1.5) *	5.1 (4.8, 5.5) *	−0.2 (−0.6, 0.3)	2.1 (1.9, 2.2) *
Central Latin America	0.153 (0.092, 0.228)	0.153 (0.105, 0.219)	0%	3.4 (2.8, 4) *	−3.5 (−7, 0.2)	−1 (−1.3, −0.7) *	0.2 (−0.4, 0.7)
Tropical Latin America	0.133 (0.072, 0.224)	0.119 (0.075, 0.174)	−10.53%	1.9 (1.7, 2.2) *	−4.8 (−5.5, −4.1) *	0.7 (−2.7, 4.3)	−0.3 (−0.8, 0.1) *
Caribbean	0.097 (0.043, 0.183)	0.205 (0.095, 0.391)	111.34%	4.1 (3.8, 4.3) *	2.3 (1.4, 3.3) *	−2 (−4.1, 0.2)	2.7 (2.3, 3.1) *
Eastern Europe	0.074 (0.053, 0.1)	0.047 (0.033, 0.065)	−36.49%	1.8 (0.1, 3.6) *	−2.2 (−2.5, −1.8) *	−4.1 (−6.3, −1.9) *	−1.7 (−2.2, −1.2) *
Southeast Asia	0.149 (0.074, 0.273)	0.156 (0.098, 0.233)	4.70%	1.3 (1.2, 1.3) *	−2.4 (−2.6, −2.2) *	0.9 (−0.7, 2.5)	0.1 (0, 0.2) *
Western Europe	0.024 (0.017, 0.034)	0.011 (0.007, 0.016)	−54.17%	−3.6 (−4.1, −3.1) *	−1.9 (−2.4, −1.4) *	−3 (−3.7, −2.4) *	−2.8 (−3.1, −2.5) *
Southern Latin America	0.099 (0.029, 0.207)	0.059 (0.016, 0.125)	−40.40%	−0.7 (−0.9, −0.5) *	−3.3 (−3.6, −3.1) *	−0.2 (−2.2, 1.9)	−1.8 (−2.1, −1.6) *
High-income Asia Pacific	0.019 (0.009, 0.032)	0.009 (0.005, 0.016)	−52.63%	−1.8 (−2.3, −1.3) *	−6.5 (−7.3, −5.7) *	0.2 (−0.5, 0.9)	−2.7 (−3, −2.3) *
Andean Latin America	0.303 (0.125, 0.55)	0.294 (0.143, 0.514)	−2.97%	−0.9 (−1, −0.7) *	1.1 (1, 1.2) *	−1.1 (−1.4, −0.7) *	−0.1 (−0.2, 0) *
Oceania	0.017 (0.004, 0.05)	0.025 (0.006, 0.067)	47.06%	1.7 (0.3, 3.2) *	3.8 (1.8, 5.9) *	0.7 (0.5, 0.9) *	1.4 (1, 1.8) *
East Asia	0.014 (0.006, 0.026)	0.023 (0.017, 0.029)	64.29%	4.1 (3.5, 4.6) *	−0.5 (−2, 1.2)	1.3 (0.9, 1.7) *	1.9 (1.5, 2.3) *
North Africa and Middle East	0.109 (0.071, 0.153)	0.109 (0.077, 0.148)	0%	1.7 (1.5, 2) *	−0.7 (−0.8, −0.5) *	−2.4 (−3.3, −1.5) *	−0.1 (−0.2, 0.1) *
South Asia	0.207 (0.075, 0.423)	0.349 (0.234, 0.485)	68.60%	1.7 (1.5, 1.9) *	5.6 (−1.5, 13.2)	0.2 (−1.4, 1.7)	1.8 (1.1, 2.6) *
Central Sub-Saharan Africa	0.09 (0.023, 0.245)	0.185 (0.081, 0.362)	105.56%	0.9 (0.7, 1) *	7.3 (6.3, 8.2) *	2.6 (1.7, 3.6) *	2.5 (2.2, 2.7) *
Eastern Sub-Saharan Africa	0.087 (0.027, 0.215)	0.174 (0.081, 0.329)	100%	0.1 (−1.6, 1.9)	3.6 (3.4, 3.8) *	−0.2 (−1.4, 1.1)	2.5 (2.1, 2.8) *
High-income North America	0.029 (0.023, 0.036)	0.015 (0.011, 0.02)	−48.28%	−0.8 (−1.1, −0.6) *	−6 (−7.8, −4.2) *	−1.9 (−2.5, −1.2) *	−2 (−2.4, −1.7) *
Southern Sub-Saharan Africa	0.238 (0.14, 0.359)	0.358 (0.235, 0.523)	50.42%	1.6 (1.2, 2.1) *	4.6 (4.3, 5) *	−2 (−2.3, −1.7) *	1.5 (1.3, 1.7) *
Western Sub-Saharan Africa	0.25 (0.09, 0.573)	0.488 (0.268, 0.809)	95.20%	2 (1.9, 2.2) *	6.3 (3.8, 8.8) *	0.6 (−0.5, 1.7)	2.4 (2, 2.7) *

Note: ASMR: age-standardized mortality rate; SDI: socio-demographic index; APCs: annual percentage changes; AAPCs: average annual percentage changes. The APCs in each stage represents the change trends between two different time points. The content in brackets represents the 95% confidence interval. * The *p*-value is less than 0.05.

**Table 2:** Age-standardized disability-adjusted life years rate, changes in the percentage, annual percentage changes, and average annual percentage changes in PM2.5-related neonatal infections from 1990 to 2019 globally and in different regions.

Location	ASDR in 1990	ASDR in 2019	Changes in Percentage 1990–2019	APCs (Stage1)	APCs (Stage2)	APCs (Stage3)	AAPCs from 1990 to2019
Global	11 (5.751, 19.44)	19.063 (12.584, 27.518)	73.30%	2.1 (1.1, 3.1) *	4 (−1.9, 10.3)	1.6 (1.5, 1.8) *	2 (1.3, 2.6) *
Low SDI	11.891 (3.667, 29.626)	21.379 (10.875, 38.727)	79.79%	1.9 (1.8, 2.1) *	6.3 (2.9, 9.8) *	0 (−1.4, 1.5)	2.2 (1.7, 2.7) *
Low-middle SDI	15.191 (5.902, 31.425)	30.059 (19.197, 43.004)	97.87%	2.4 (2.2, 2.5) *	5.1 (−1, 11.6)	0.8 (−0.5, 2.1)	2.4 (1.7, 3) *
Middle SDI	10.825 (6.925, 15.505)	16.278 (12.352, 20.858)	50.37%	3.1 (2.9, 3.4) *	1 (0.6, 1.3) *	−0.6 (−1, −0.1) *	1.4 (1.2, 1.5) *
High-middle SDI	7.168 (5.336, 9.433)	6.506 (5.099, 8.179)	−9.24%	2.2 (1.9, 2.5) *	−1.3 (−1.8, −0.9) *	−2.5 (−2.9, −2.1) *	−0.4 (−0.6, −0.2) *
High SDI	3.257 (2.382, 4.544)	1.976 (1.451, 2.661)	−39.33%	2.4 (2.1, 2.7) *	−5.4 (−5.8, −5) *	−2.7 (−3.1, −2.2) *	−1.7 (−2, −1.5) *
Central Europe	4.221 (2.716, 5.8)	1.29 (0.895, 1.818)	−69.44%	−0.7 (−1.2, −0.1) *	−7.7 (−8.1, −7.3) *	−0.9 (−3.6, 1.8)	−4.2 (−4.6, −3.7) *
Australasia	1.563 (0.121, 4.236)	0.495 (0.108, 1.044)	−68.33%	−1.1 (−5.1, 3.2)	−9.9 (−21.1, 2.8)	−3.7 (−4, −3.4) *	−4 (−5.4, −2.7) *
Central Asia	4.72 (2.462, 7.608)	8.472 (5.094, 12.503)	79.49%	1.3 (1.2, 1.4) *	5.2 (4.9, 5.5) *	−0.2 (−0.5, 0.2)	2.1 (1.9, 2.2) *
Central Latin America	13.615 (8.164, 20.249)	13.632 (9.315, 19.506)	0.12%	3.4 (2.8, 4) *	−3.4 (−6.9, 0.2)	−1 (−1.3, −0.7) *	0.2 (−0.4, 0.7)
Tropical Latin America	11.842 (6.356, 19.914)	10.612 (6.657, 15.437)	−10.39%	1.9 (1.7, 2.2) *	−4.8 (−5.5, −4) *	0.7 (−2.8, 4.3)	−0.3 (−0.8, 0.1) *
Caribbean	8.638 (3.805, 16.258)	18.209 (8.494, 34.722)	110.80%	4 (3.8, 4.3) *	2.3 (1.4, 3.3) *	−2 (−4.1, 0.1)	2.7 (2.3, 3.1) *
Eastern Europe	6.545 (4.727, 8.871)	4.181 (2.926, 5.739)	−36.12%	1.9 (0.2, 3.7) *	−2.2 (−2.5, −1.8) *	−4.1 (−6.2, −1.9) *	−1.7 (−2.2, −1.2) *
Southeast Asia	13.275 (6.574, 24.29)	13.837 (8.679, 20.725)	4.23%	1.2 (1.2, 1.3) *	−2.4 (−2.6, −2.2) *	0.8 (−0.8, 2.4)	0.1 (−0.1, 0.2) *
Western Europe	2.175 (1.494, 3.024)	0.967 (0.612, 1.435)	−55.54%	−4.1 (−4.4, −3.8) *	−2.2 (−2.3, −2.1) *	−3.2 (−3.7, −2.7) *	−2.8 (−2.9, −2.7) *
Southern Latin America	8.765 (2.561, 18.419)	5.227 (1.422, 11.08)	−40.37%	−0.7 (−0.9, −0.5) *	−3.3 (−3.5, −3.1) *	−0.3 (−2.3, 1.7)	−1.8 (−2.1, −1.6) *
High-income Asia Pacific	1.682 (0.825, 2.866)	0.835 (0.454, 1.379)	−50.36%	−1.4 (−1.7, −1) *	−6.2 (−6.5, −5.8) *	0.4 (0, 0.8)	−2.5 (−2.7, −2.3) *
Andean Latin America	26.968 (11.091, 48.951)	26.173 (12.766, 45.663)	−2.95%	−0.9 (−1, −0.7) *	1.1 (1, 1.2) *	−1.1 (−1.4, −0.7) *	−0.1 (−0.2, 0) *
Oceania	1.509 (0.35, 4.43)	2.239 (0.518, 5.947)	48.38%	1.5 (0.6, 2.4) *	4 (2.7, 5.3) *	0.7 (0.6, 0.8) *	1.4 (1.1, 1.6) *
East Asia	1.271 (0.577, 2.286)	2.067 (1.56, 2.619)	62.63%	3.9 (3.4, 4.4) *	−0.2 (−1.2, 0.8)	1.3 (1, 1.7) *	1.8 (1.5, 2.2) *
North Africa and Middle East	9.721 (6.303, 13.617)	9.657 (6.869, 13.193)	−0.66%	2 (1.7, 2.2) *	−0.6 (−0.7, −0.5) *	−2.5 (−3.3, −1.7) *	−0.1 (−0.2, 0.1) *
South Asia	18.356 (6.627, 37.552)	31.035 (20.843, 43.113)	69.07%	1.7 (1.5, 1.9) *	4.6 (1.1, 8.3) *	0.3 (−1.2, 1.9)	1.8 (1.3, 2.4) *
Central Sub-Saharan Africa	7.979 (2.046, 21.759)	16.41 (7.236, 32.129)	105.66%	0.9 (0.8, 1) *	7.2 (6.3, 8.2) *	2.6 (1.7, 3.5) *	2.5 (2.2, 2.7) *
Eastern Sub-Saharan Africa	7.696 (2.436, 19.135)	15.487 (7.207, 29.262)	101.23%	0.2 (−1.6, 1.9)	3.6 (3.4, 3.8) *	−0.2 (−1.4, 1.1)	2.5 (2.1, 2.8) *
High income North America	2.592 (2.014, 3.191)	1.369 (1.017, 1.791)	−47.18%	−0.7 (−0.9, −0.5) *	−5.2 (−6.2, −4.1) *	−2 (−2.5, −1.5) *	−2 (−2.3, −1.7) *
Southern Sub-Saharan Africa	21.147 (12.473, 31.888)	31.855 (20.848, 46.494)	50.64%	1.7 (1.2, 2.1) *	4.6 (4.3, 4.9) *	−2 (−2.3, −1.7) *	1.5 (1.3, 1.7) *
Western Sub-Saharan Africa	22.198 (7.975, 50.892)	43.399 (23.792, 71.924)	95.51%	2.1 (1.9, 2.2) *	6.3 (3.8, 8.8) *	0.6 (−0.5, 1.6)	2.4 (2, 2.7) *

Note: ASDR: age-standardized disability-adjusted life years rate; SDI: socio-demographic index; APCs: annual percentage changes; AAPCs: average annual percentage changes. The APCs in each stage represent the change trends between two different time points. The content in brackets represents the 95% confidence interval. * The *p*-value is less than 0.05.

## Data Availability

Data are available in a publicly accessible repository. Publicly available datasets were analyzed in this study. This data can be found using the Global Health Data Exchange (GHDx, http://ghdx.healthdata.org/gbd-results-tool, accessed on 4 January 2022).

## Supplementary Materials

The following supporting information can be downloaded at: https://www.mdpi.com/article/10.3390/ijerph19095399/s1, Figures S1–S21: Trends in the age-standardized mortality rate of PM2.5-related neonatal infections in 21 regions from 1990 to 2019; Figures S22–S42: Trends in the age-standardized disability-adjusted life rate of PM2.5-related neonatal infections in 21 regions from 1990 to 2019; Figure S43: The changes of age-standardized disability-adjusted life years rate in PM2.5-related neonatal infections in the global and various socio-demographic index (SDI) regions from 1990 to 2019; Figure S44: Age-standardized disability-adjusted life years rates (ASDR) of PM2.5-related neonatal infections in the global and 21 regions in 1990–2019. The expected value is represented by the solid blue line. The trends between ASDR and the socio-demographic index are represented by the Pearson correlation coefficient and *p*-value in the picture. The ASDR of PM2.5-related neonatal infections in four regions (South Asia, Southern Sub-Saharan Africa, Western Sub-Saharan Africa, Andean Latin America) were obviously higher than the expected value; Figure S45: The ratio of male to female in age-standardized disability-adjusted life years rate (ASDR) for PM2.5-related neonatal infections in different socio-demographic index(SDI) regions in 1990–2019.
