# Supplementary material for: PM2.5-Related Neonatal Infections: A Global Burden Study from 1990 to 2019
Source: Int J Environ Res Public Health. 2022 Apr 28;19(9):5399. doi: 10.3390/ijerph19095399 (PMC9105282; doi:10.3390/ijerph19095399)
Supplement: Supplementary file 1 [file ijerph-19-05399-s001.zip › ijerph-1626770-supplementary.pdf]

Supplementary Material

PM2.5-Related Neonatal Infections: A Global Burden Study from 1990 to 2019

ZEYU TANG, JINZHU JIA

International Journal of Environmental Research and Public Health

The authors' institutional affiliations:

First author: ZEYU TANG, Department of Biostatistics, School of Public Health, Peking University, No.38, Xueyuan Road, Beijing, the People's Republic of China.

Corresponding author: JINZHU JIA, Department of Biostatistics, School of Public Health, Peking University, No.38, Xueyuan Road, Beijing, the People's Republic of China. E-mail: jzjia@math.pku.edu.cn

A short (about 30 words) description of the author(s):

ZEYU TANG is a graduate student at Department of Biostatistics, School of Public Health, Peking University, Beijing, China. His research areas include developing prediction models for maternal adverse outcomes, detecting high-order interactions, methods of imputing missing data and developing trajectory algorithm.

JINZHU JIA is an associate professor at Department of Biostatistics, School of Public Health, Peking University, Beijing, China. His main research areas include biostatistics, high-dimensional statistical inference, big data analysis, statistical machine learning and causal inference.

Part 1 (Figures S1–S21): Trends in the age-standardized mortality rate of PM2.5-related neonatal infections in 21 regions from 1990 to 2019.

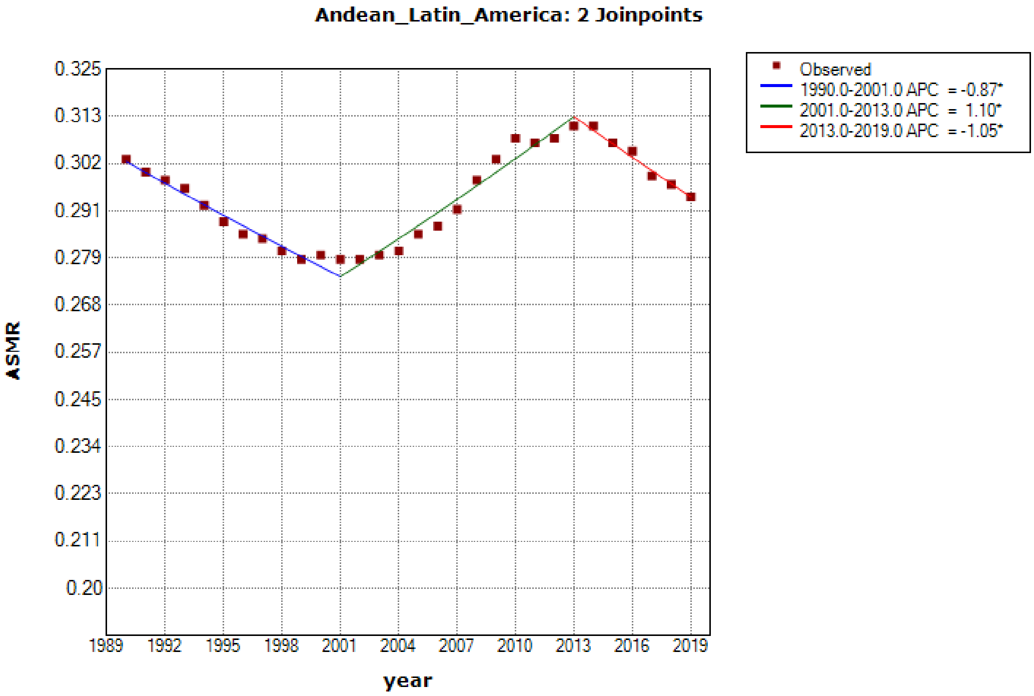

Figure S1. Trends in the age-standardized mortality rate (ASMR, per 100,000 population) of PM2.5-related neonatal infections in Andean Latin America from 1990 to 2019. $^{*}P$<0.05.

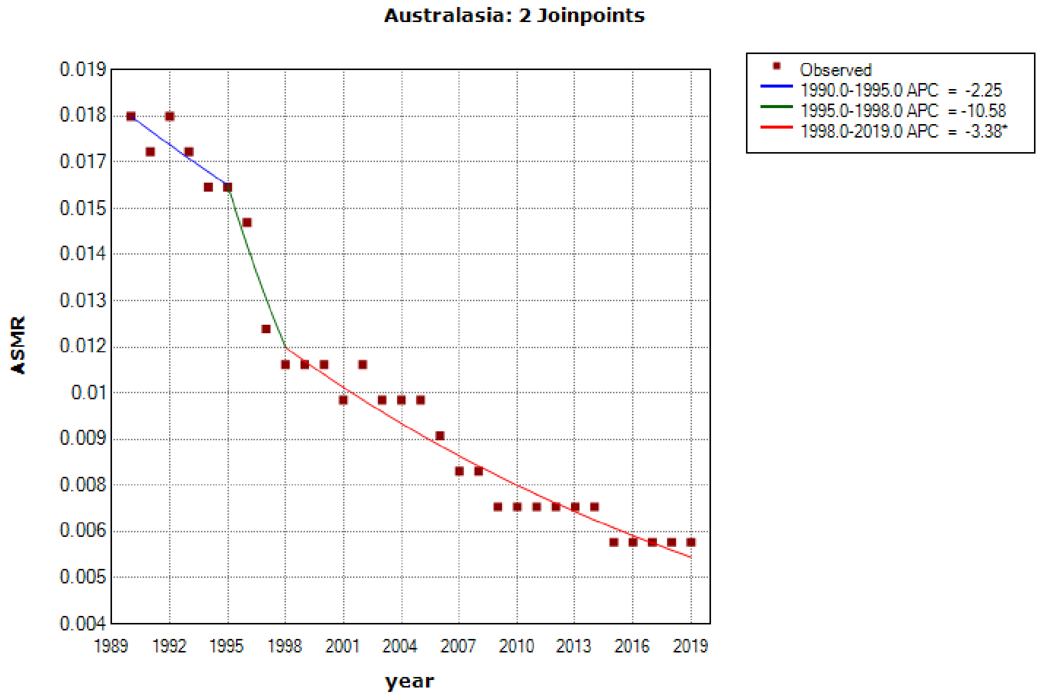

Figure S2. Trends in the age-standardized mortality rate (ASMR, per 100,000 population) of PM2.5-related neonatal infections in Australasia from 1990 to 2019. $^{*}P<0.05$.

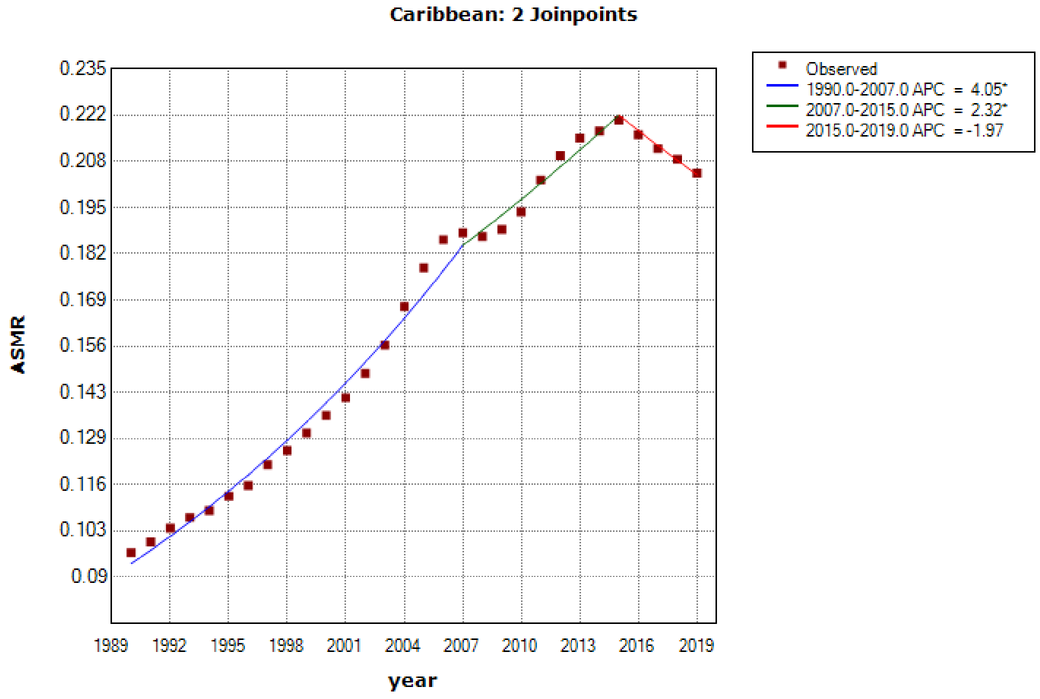

Figure S3. Trends in the age-standardized mortality rate (ASMR, per 100,000 population) of PM2.5-related neonatal infections in Caribbean from 1990 to 2019. $^{*}P<0.05$.

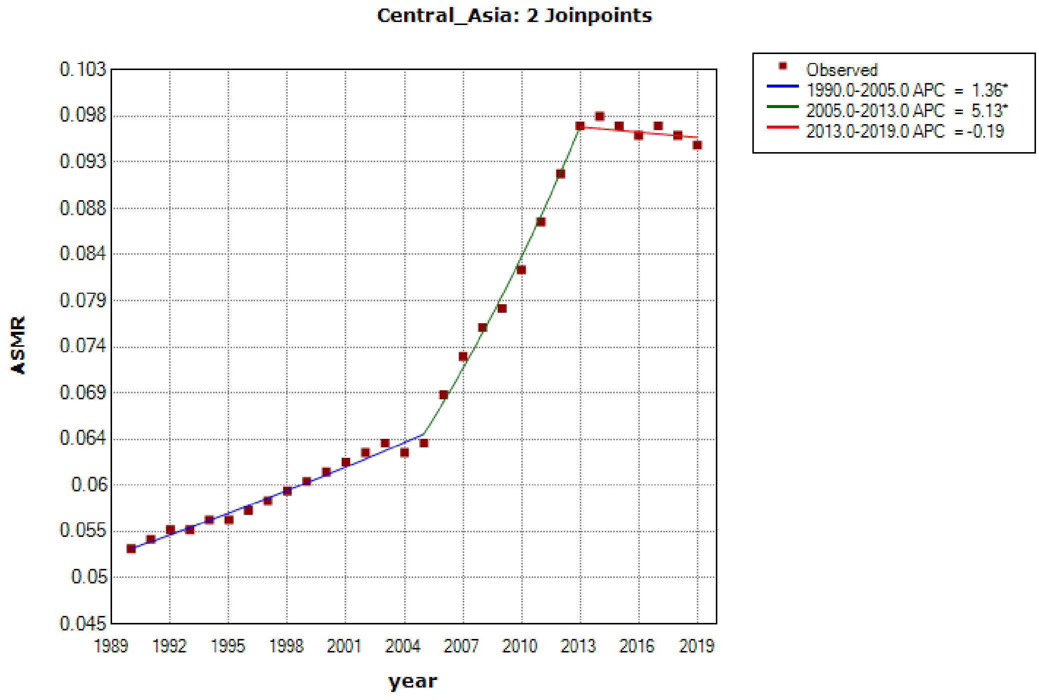

Figure S4. Trends in the age-standardized mortality rate (ASMR, per 100,000 population) of PM2.5-related neonatal infections in Central Asia from 1990 to 2019. *P<0.05.

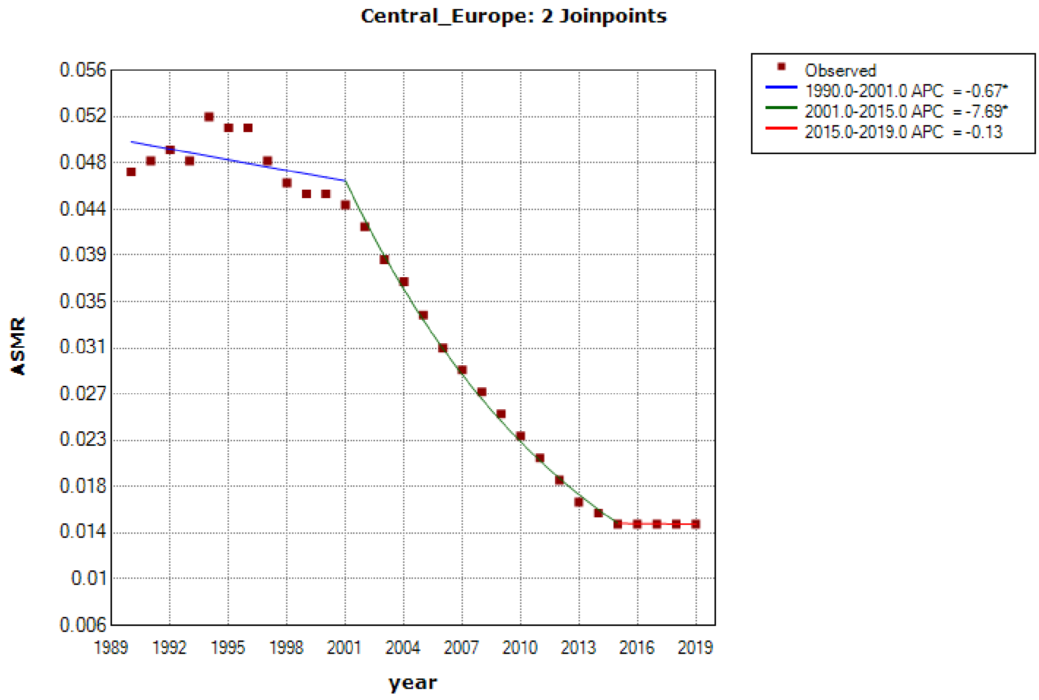

Figure S5. Trends in the age-standardized mortality rate (ASMR, per 100,000 population) of PM2.5-related neonatal infections in Central Europe from 1990 to 2019. *P<0.05.

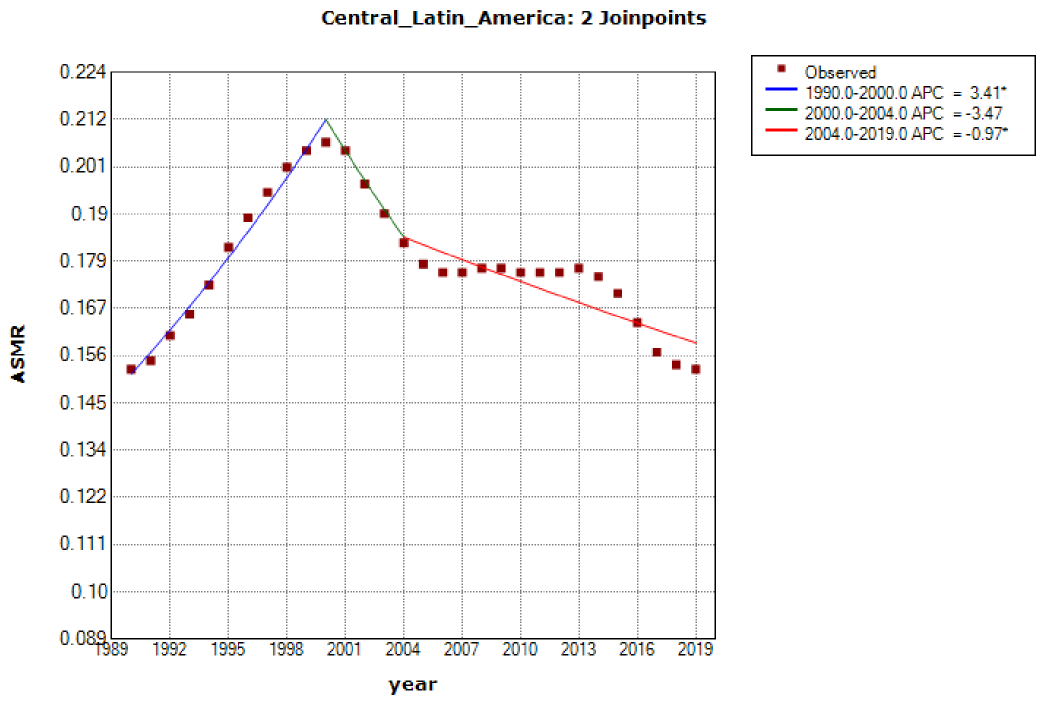

Figure S6. Trends in the age-standardized mortality rate (ASMR, per 100,000 population) of PM2.5-related neonatal infections in Central Latin America from 1990 to 2019. $^{*}P<0.05$.

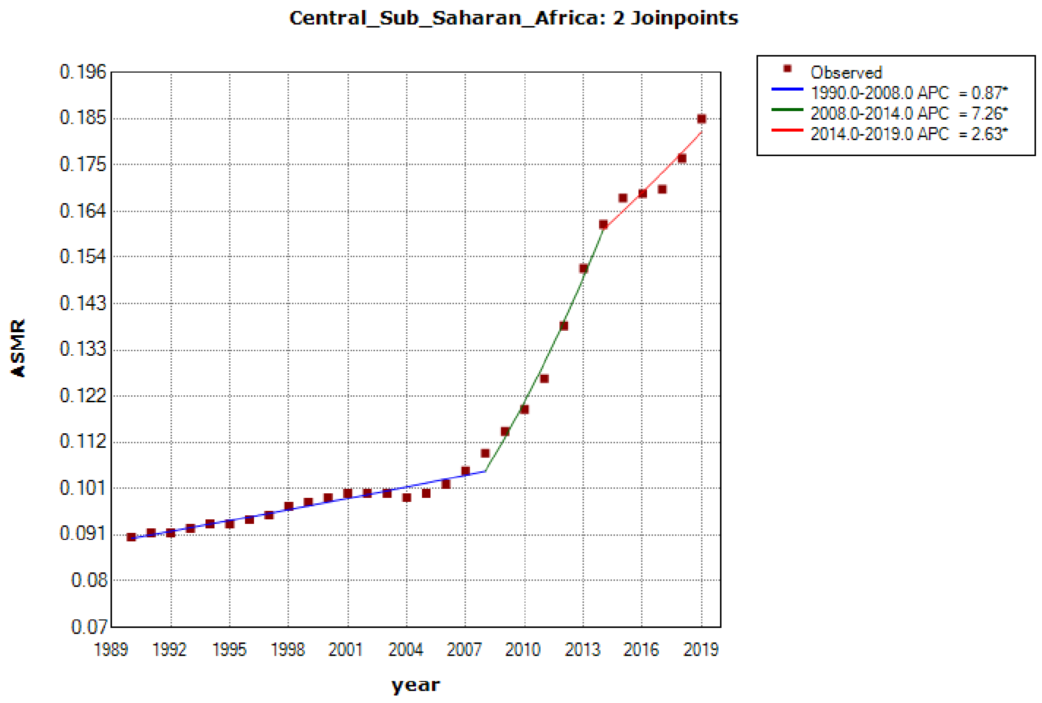

Figure S7. Trends in the age-standardized mortality rate (ASMR, per 100,000 population) of PM2.5-related neonatal infections in Central Sub Saharan Africa from 1990 to 2019. $^{*}P<0.05$.

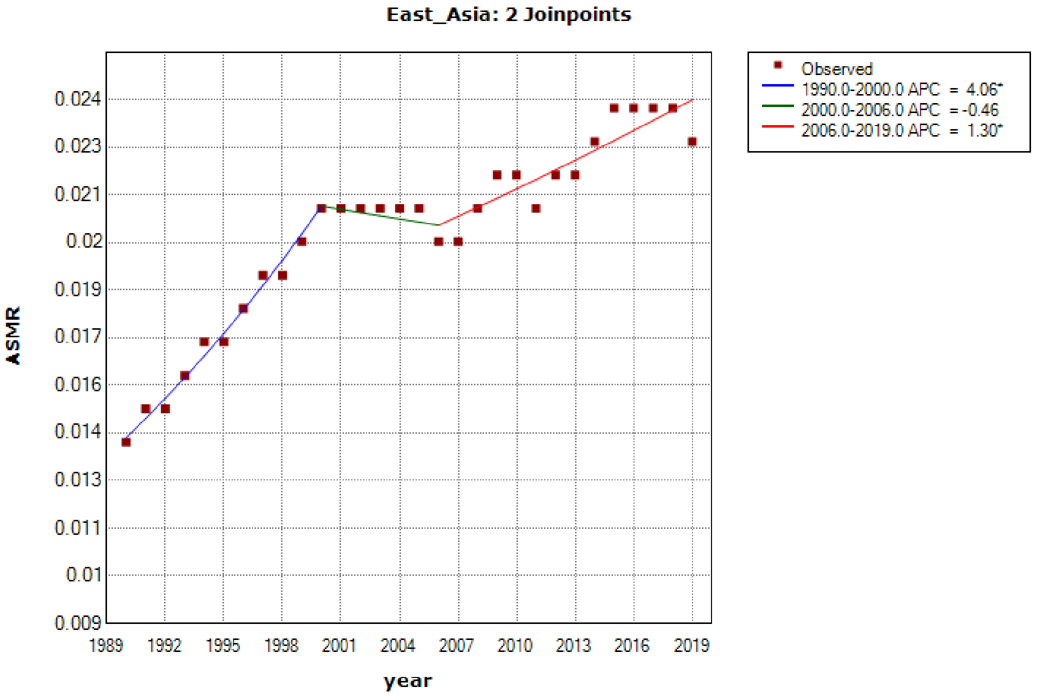

Figure S8. Trends in the age-standardized mortality rate (ASMR, per 100,000 population) of PM2.5-related neonatal infections in East Asia from 1990 to 2019. $^{*}P$<0.05.

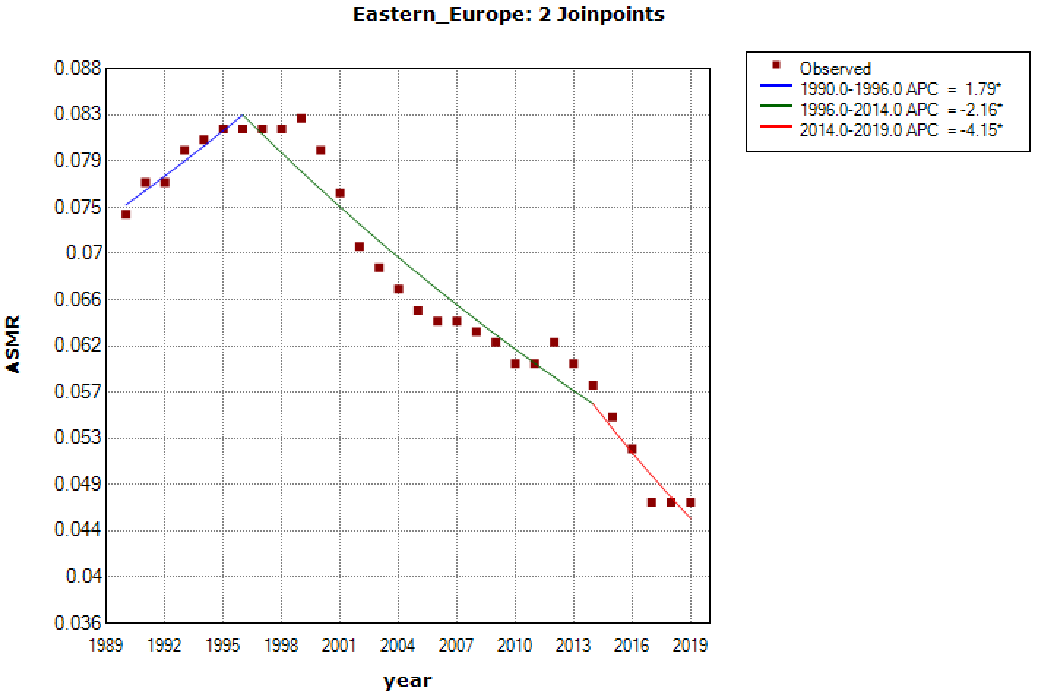

Figure S9. Trends in the age-standardized mortality rate (ASMR, per 100,000 population) of PM2.5-related neonatal infections in Eastern Europe from 1990 to 2019. $^{*}P$<0.05.

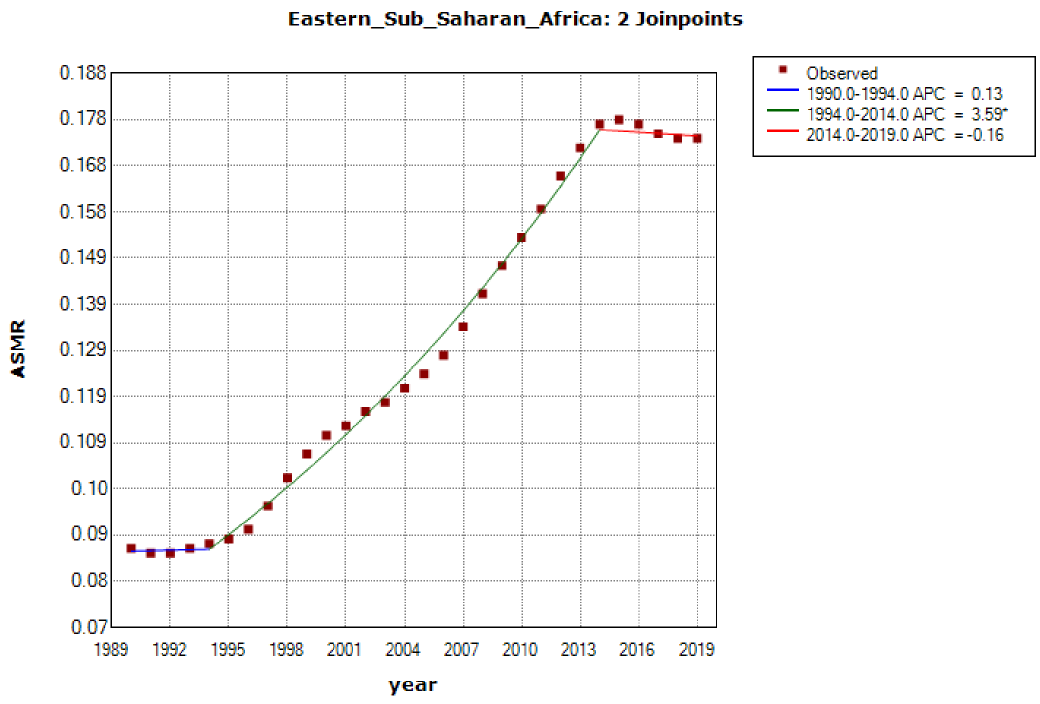

Figure S10. Trends in the age-standardized mortality rate (ASMR, per 100,000 population) of PM2.5-related neonatal infections in Eastern Sub Saharan Africa from 1990 to 2019. $^{*}P$<0.05.

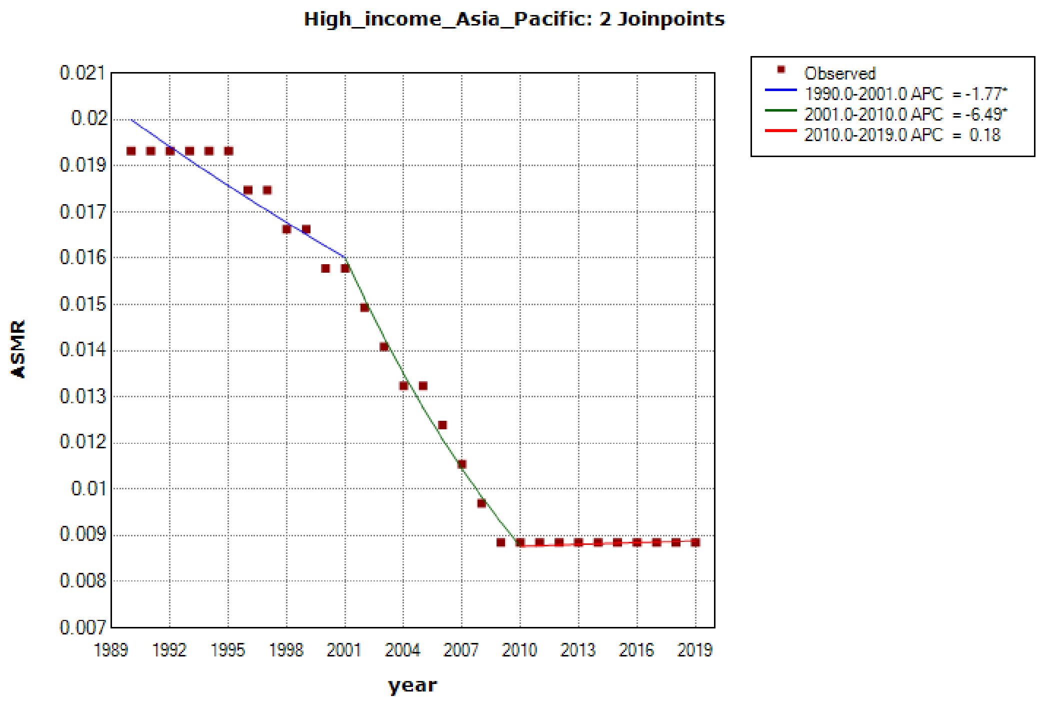

Figure S11. Trends in the age-standardized mortality rate (ASMR, per 100,000 population) of PM2.5-related neonatal infections in High income Asia Pacific from 1990 to 2019. $^{*}P$<0.05.

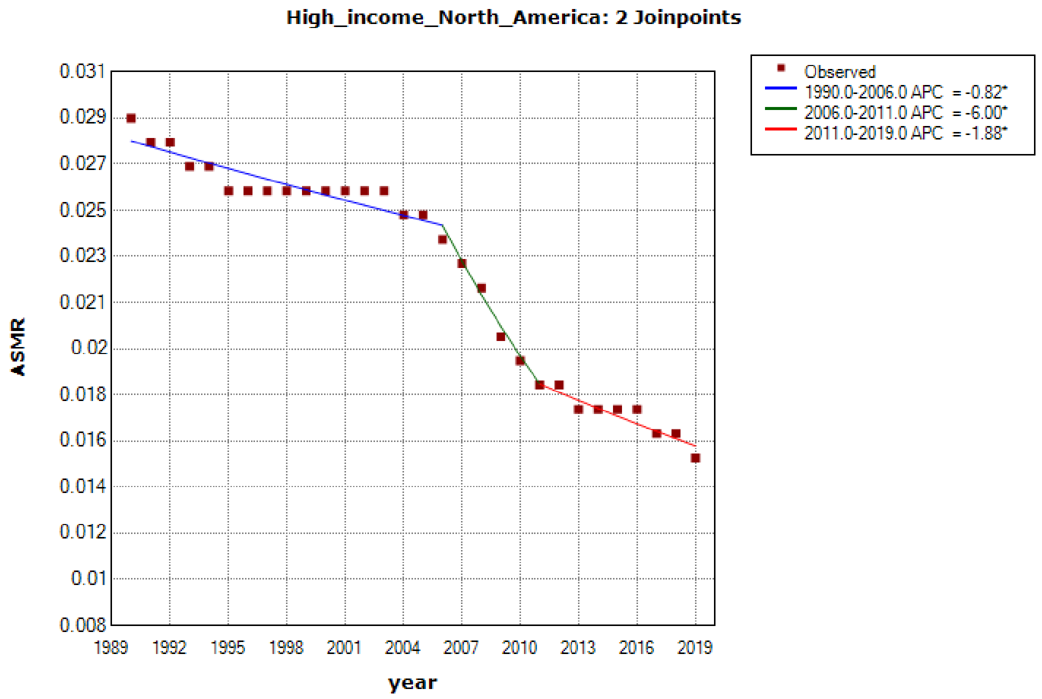

Figure S12. Trends in the age-standardized mortality rate (ASMR, per 100,000 population) of PM2.5-related neonatal infections in High income North America from 1990 to 2019. $^{*}P$<0.05.

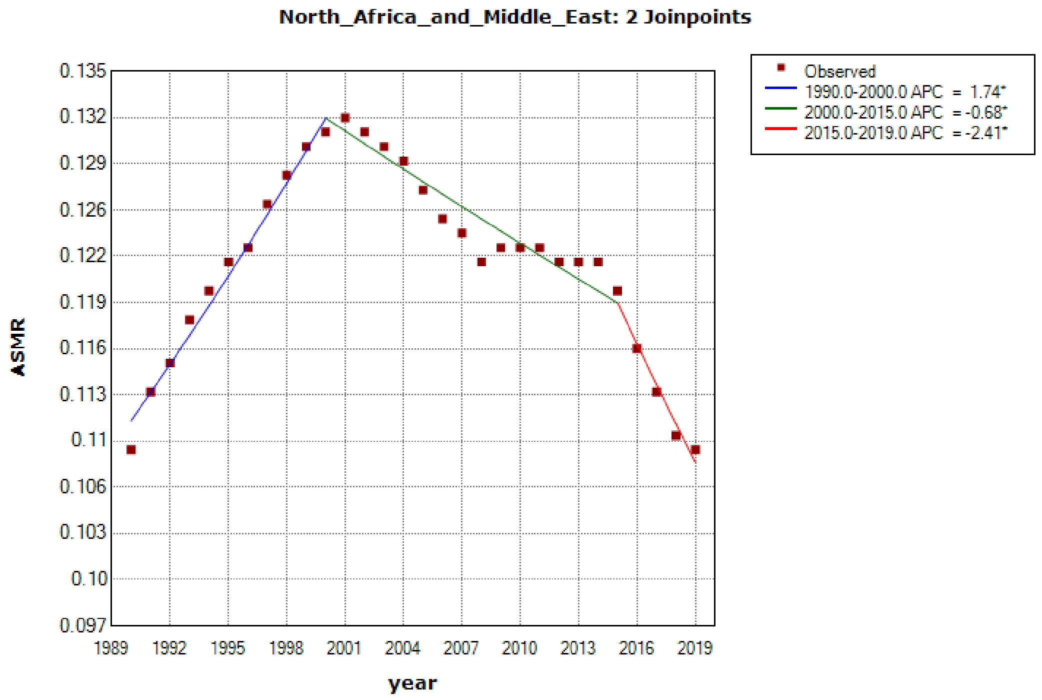

Figure S13. Trends in the age-standardized mortality rate (ASMR, per 100,000 population) of PM2.5-related neonatal infections in North Africa and Middle East from 1990 to 2019. $^{*}P$<0.05.

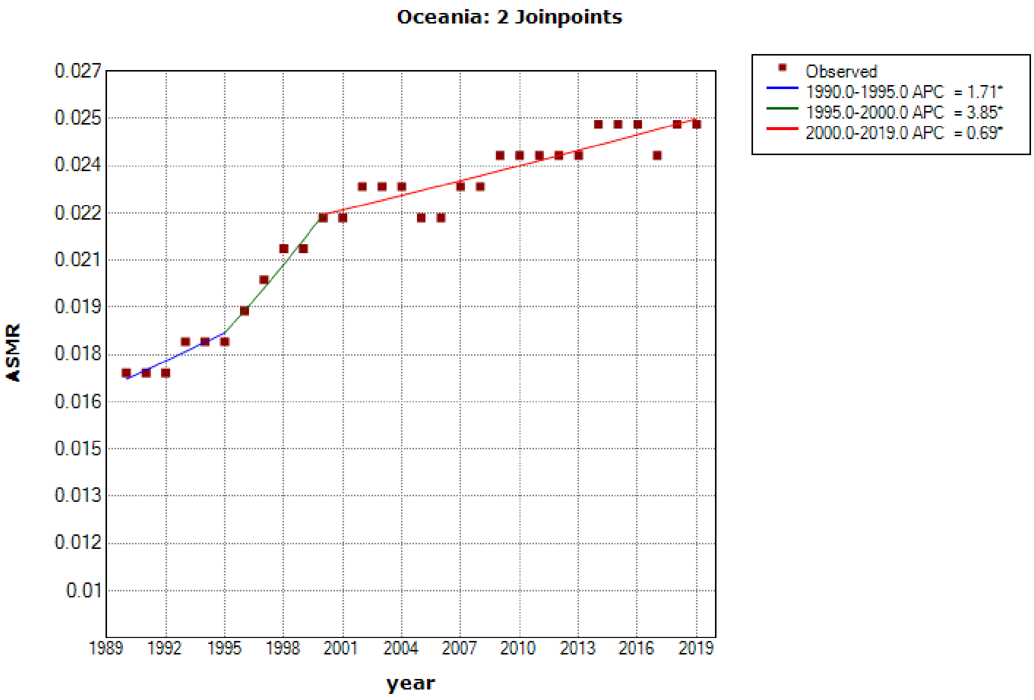

Figure S14. Trends in the age-standardized mortality rate (ASMR, per 100,000 population) of PM2.5-related neonatal infections in Oceania from 1990 to 2019. *P<0.05.

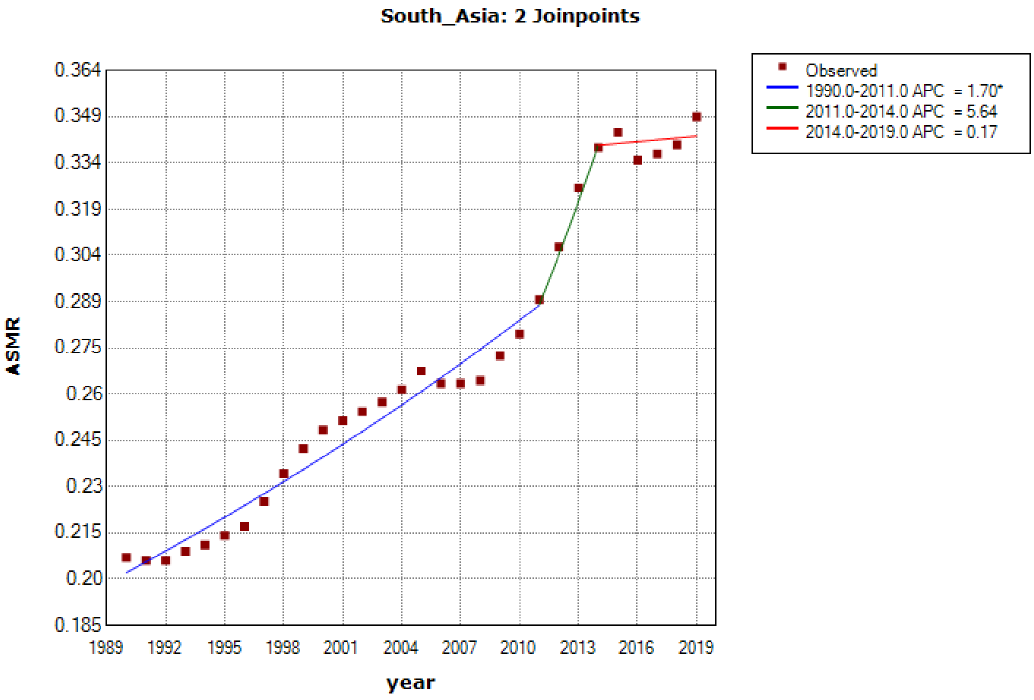

Figure S15. Trends in the age-standardized mortality rate (ASMR, per 100,000 population) of PM2.5-related neonatal infections in South Asia from 1990 to 2019. *P<0.05.

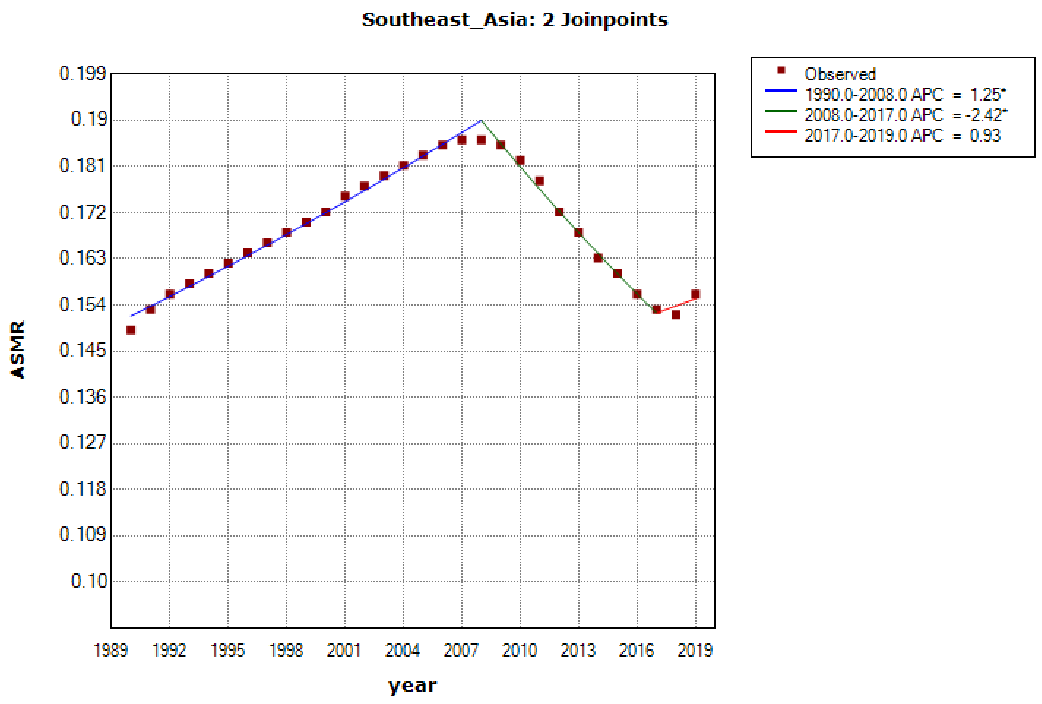

Figure S16. Trends in the age-standardized mortality rate (ASMR, per 100,000 population) of PM2.5-related neonatal infections in Southeast Asia from 1990 to 2019. *P<0.05.

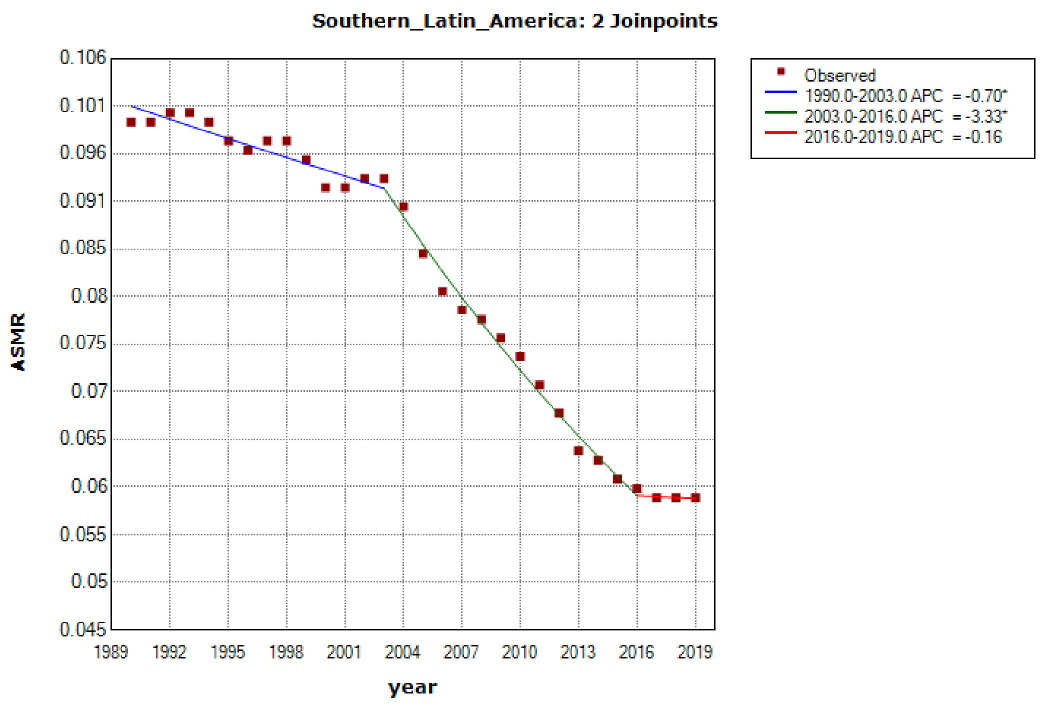

Figure S17. Trends in the age-standardized mortality rate (ASMR, per 100,000 population) of PM2.5-related neonatal infections in Southern Latin America from 1990 to 2019. *P<0.05.

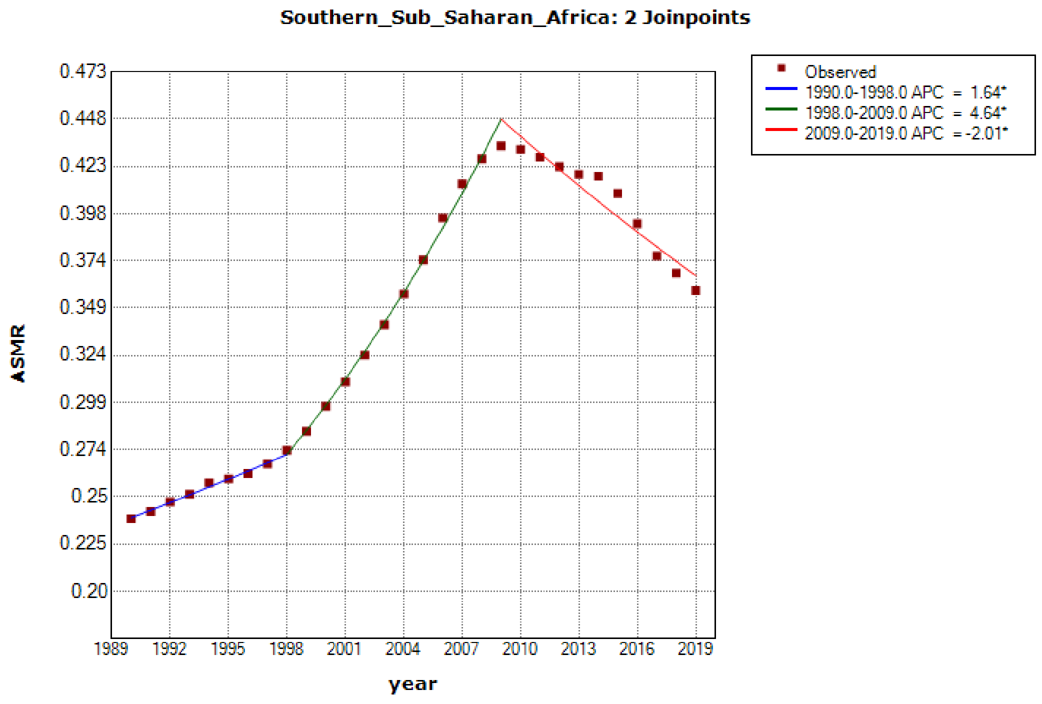

Figure S18. Trends in the age-standardized mortality rate (ASMR, per 100,000 population) of PM2.5-related neonatal infections in Southern Sub Saharan Africa from 1990 to 2019. *P<0.05.

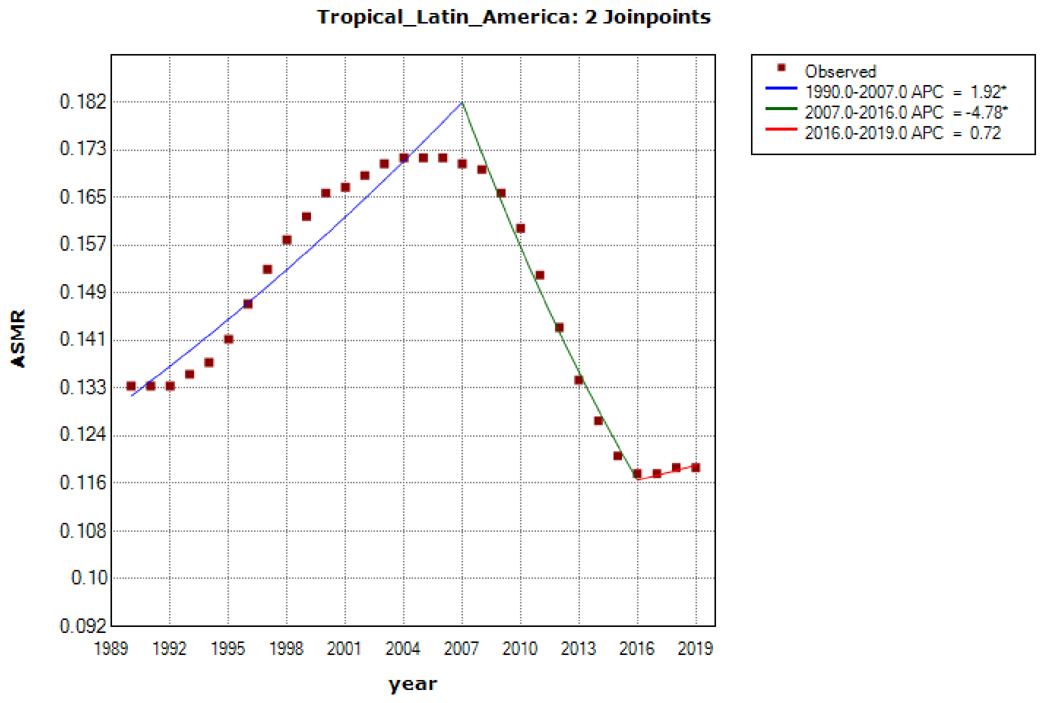

Figure S19. Trends in the age-standardized mortality rate (ASMR, per 100,000 population) of PM2.5-related neonatal infections in Tropical Latin America from 1990 to 2019. *P<0.05.

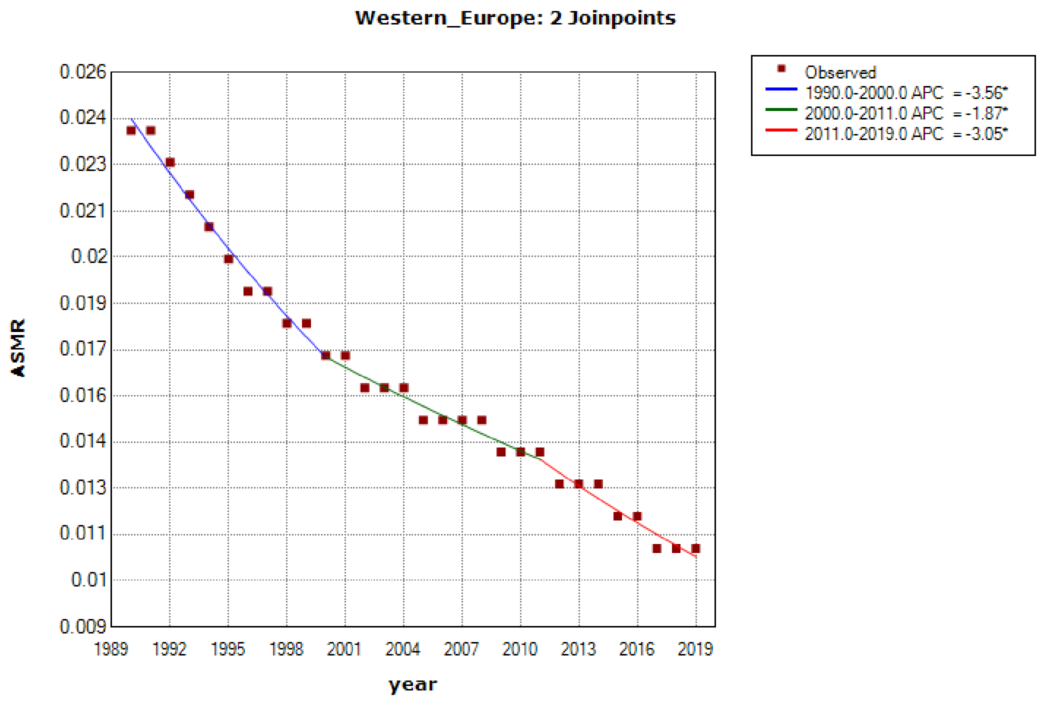

Figure S20. Trends in the age-standardized mortality rate (ASMR, per 100,000 population) of PM2.5-related neonatal infections in Western Europe from 1990 to 2019. $^{*}P$<0.05.

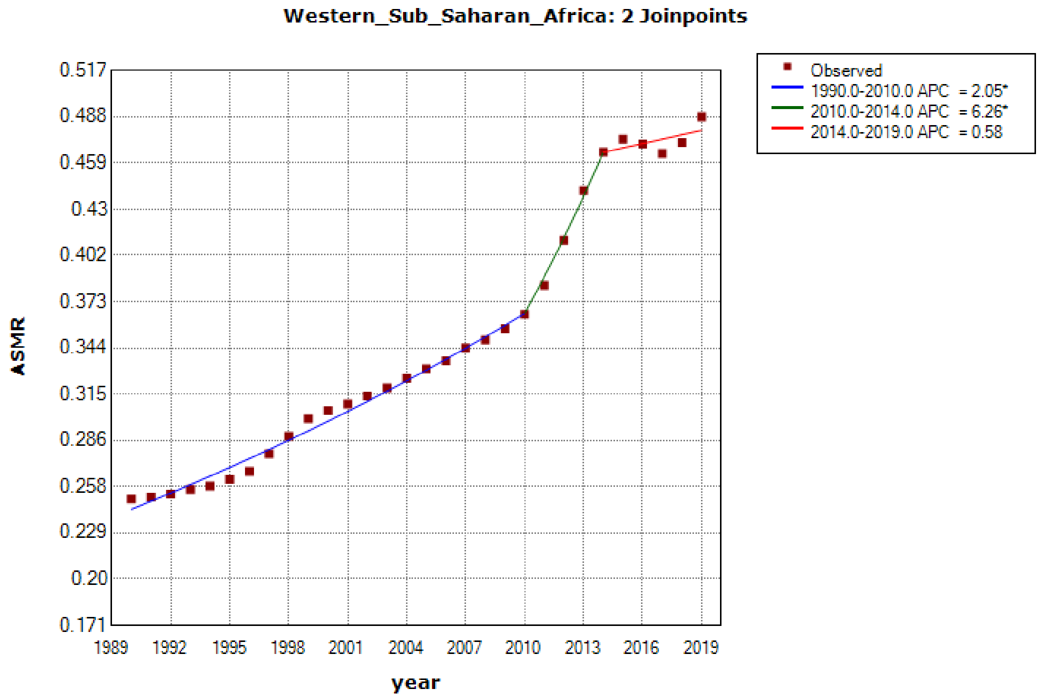

Figure S21. Trends in the age-standardized mortality rate (ASMR, per 100,000 population) of PM2.5-related neonatal infections in Western Sub Saharan Africa from 1990 to 2019. $^{*}P$<0.05.

Part 2 (Figures S22–S42): Trends in the age-standardized disability-adjusted life rate of PM2.5-related neonatal infections in 21 regions from 1990 to 2019.

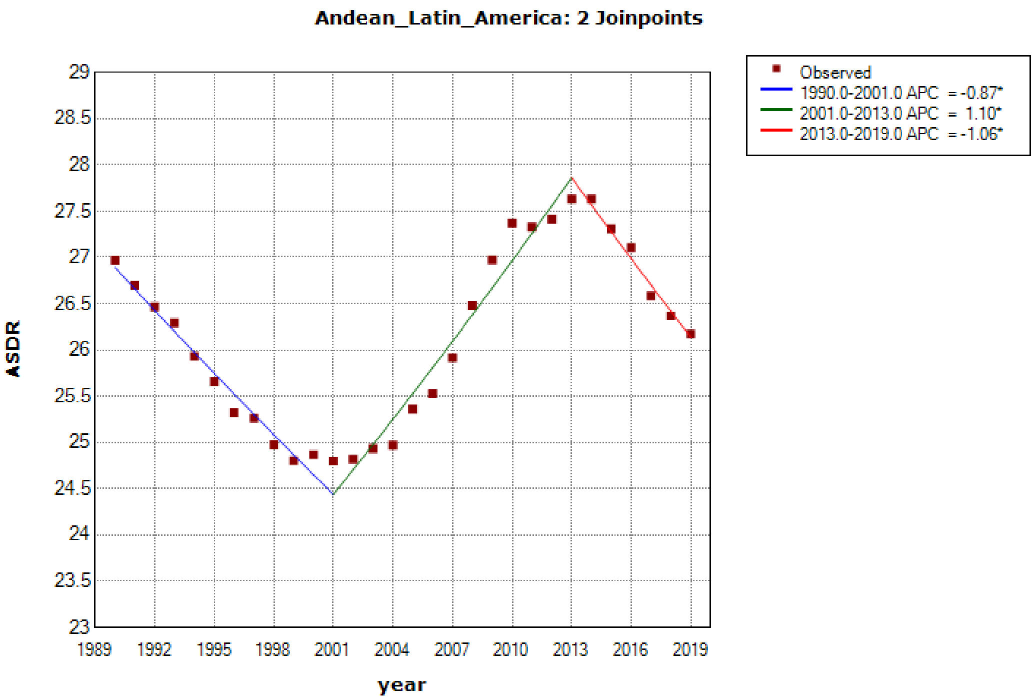

Figure S22. Trends in the age-standardized disability-adjusted life rate (ASDR, per 100,000 population) of PM2.5-related neonatal infections in Andean Latin America from 1990 to 2019. *P<0.05.

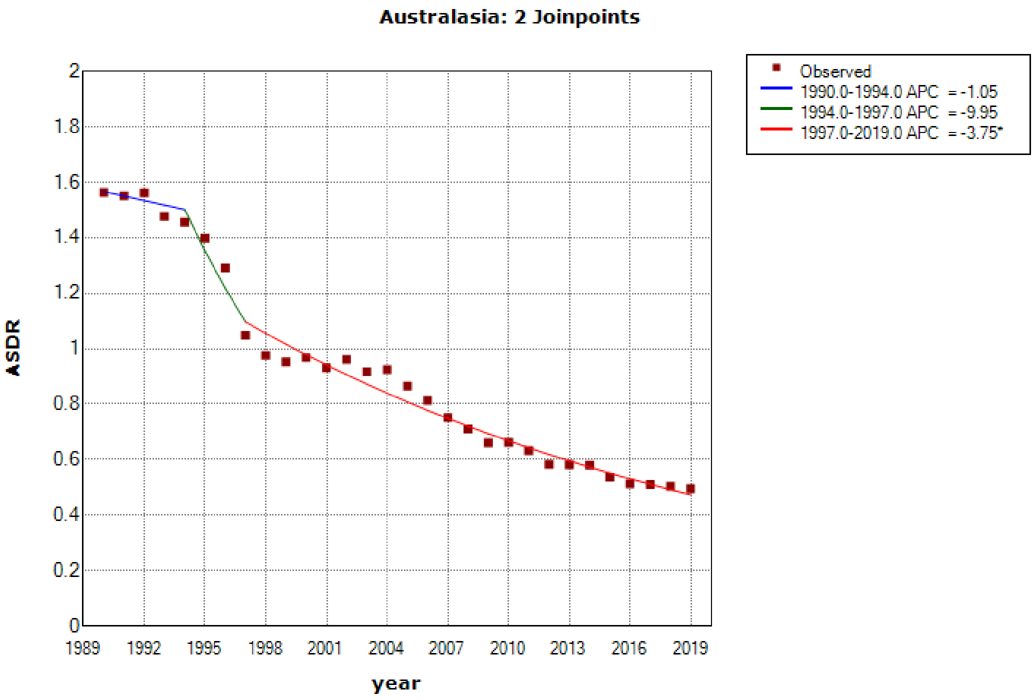

Figure S23. Trends in the age-standardized disability-adjusted life rate (ASDR, per 100,000 population) of PM2.5-related neonatal infections in Australasia from 1990 to 2019. *P<0.05.

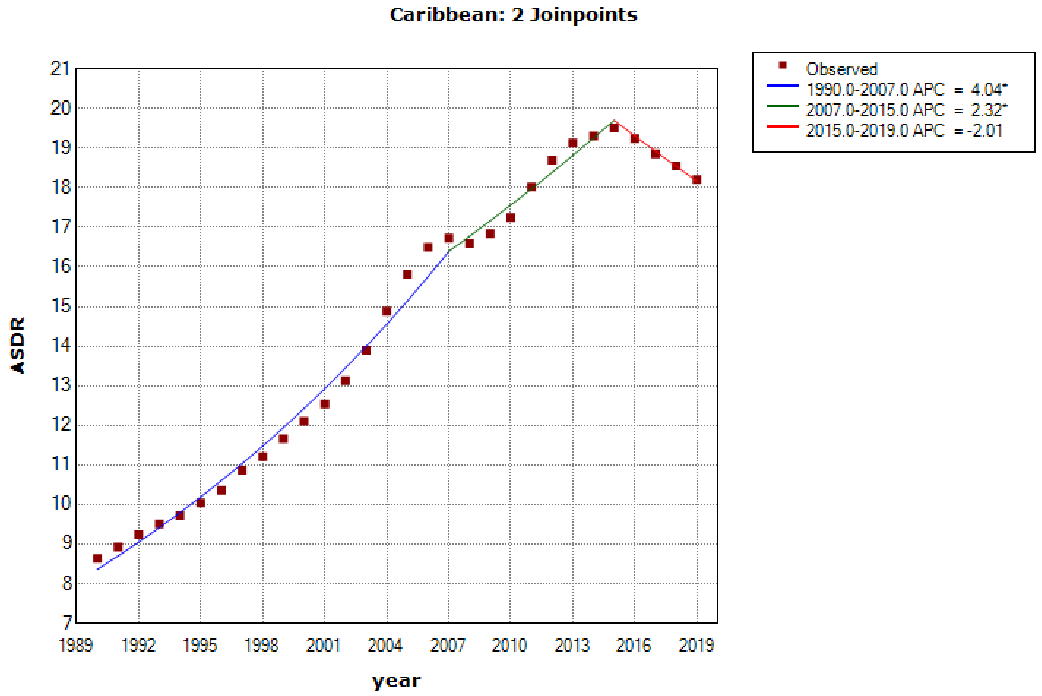

Figure S24. Trends in the age-standardized disability-adjusted life rate (ASDR, per 100,000 population) of PM2.5-related neonatal infections in Caribbean from 1990 to 2019. $^{*}P<0.05$.

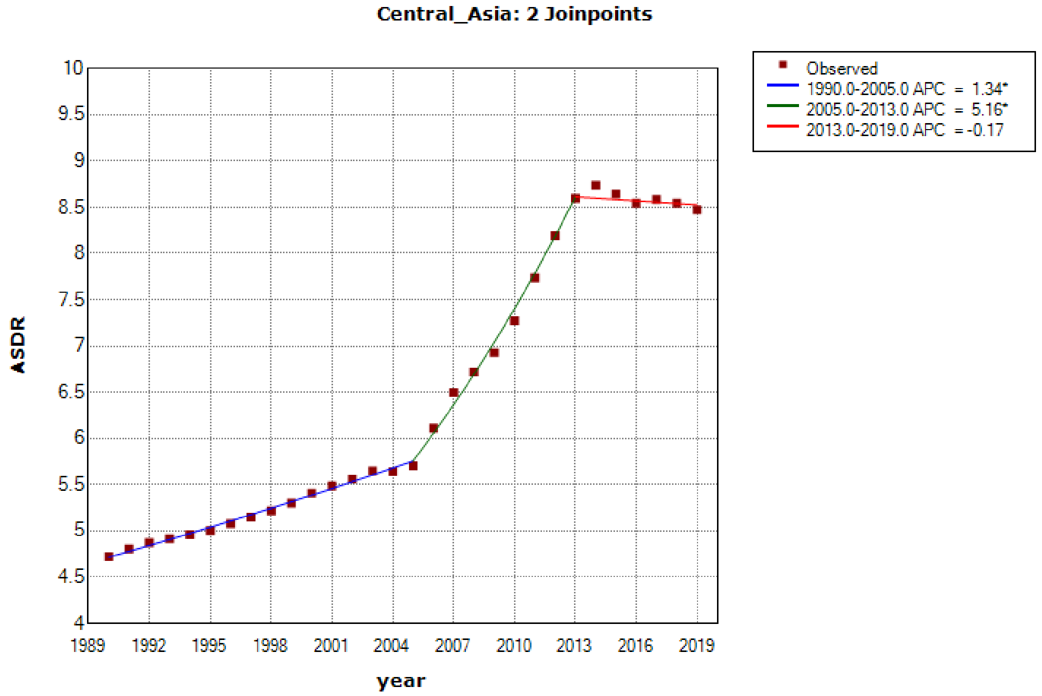

Figure S25. Trends in the age-standardized disability-adjusted life rate (ASDR, per 100,000 population) of PM2.5-related neonatal infections in Central Asia from 1990 to 2019. $^{*}P<0.05$.

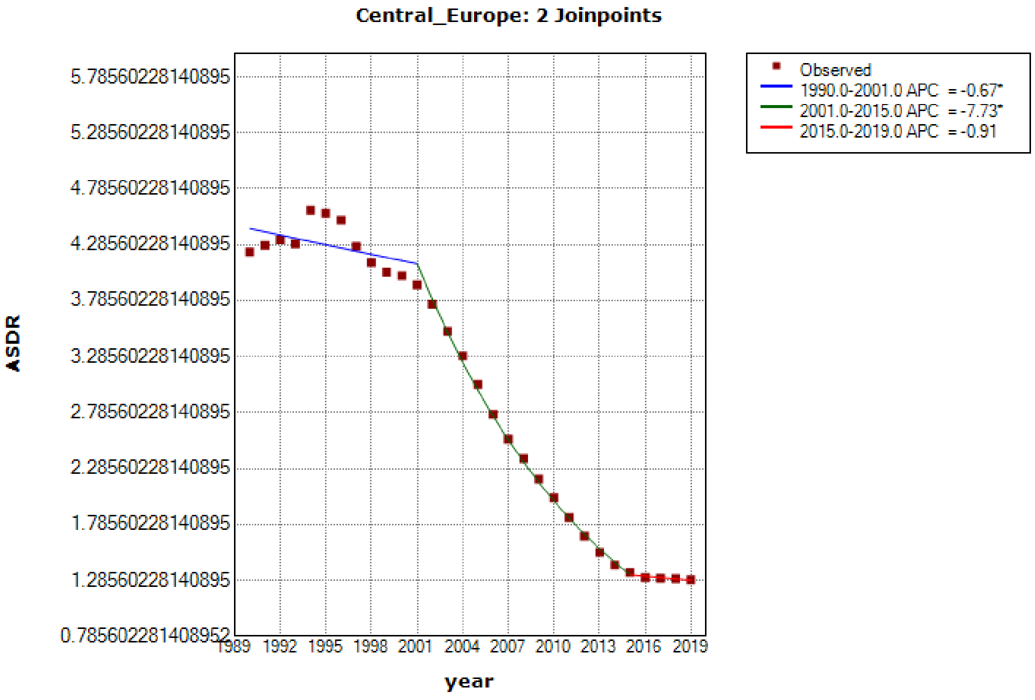

Figure S26. Trends in the age-standardized disability-adjusted life rate (ASDR, per 100,000 population) of PM2.5-related neonatal infections in Central Europe from 1990 to 2019. $^{*}P$<0.05.

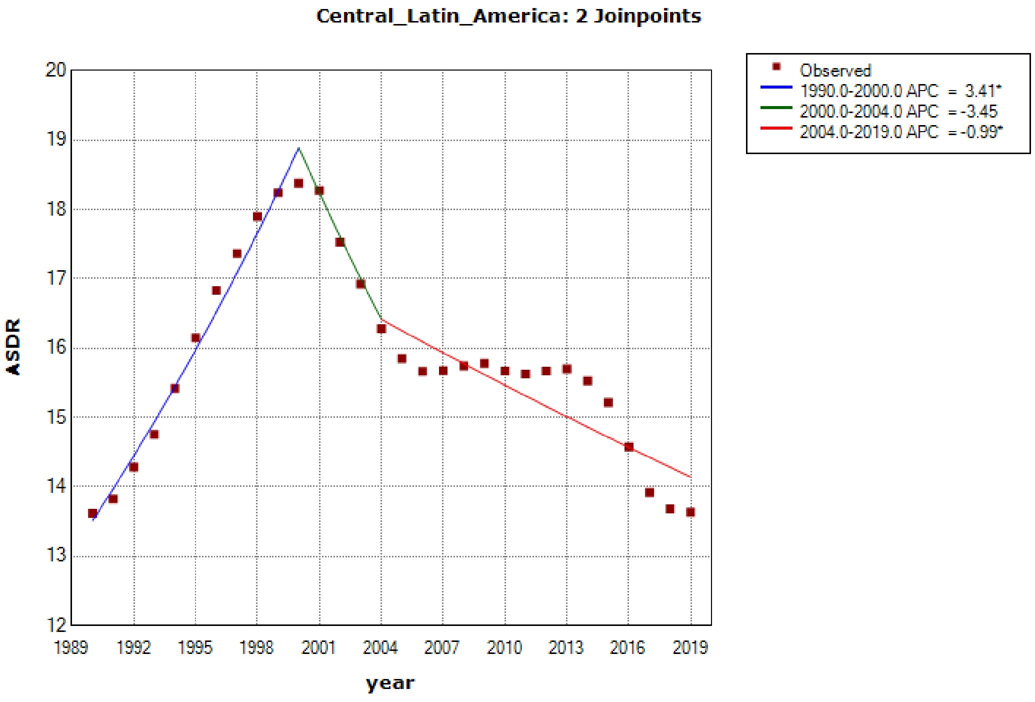

Figure S27. Trends in the age-standardized disability-adjusted life rate (ASDR, per 100,000 population) of PM2.5-related neonatal infections in Central Latin America from 1990 to 2019. $^{*}P$<0.05.

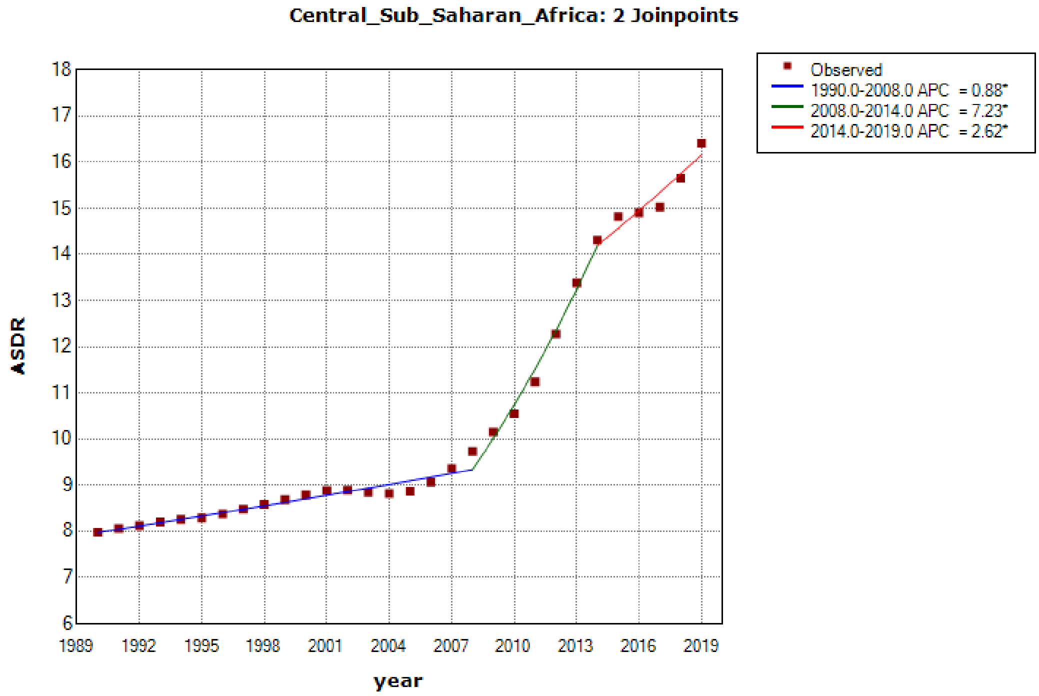

Figure S28. Trends in the age-standardized disability-adjusted life rate (ASDR, per 100,000 population) of PM2.5-related neonatal infections in Central Sub Saharan Africa from 1990 to 2019. *P<0.05.

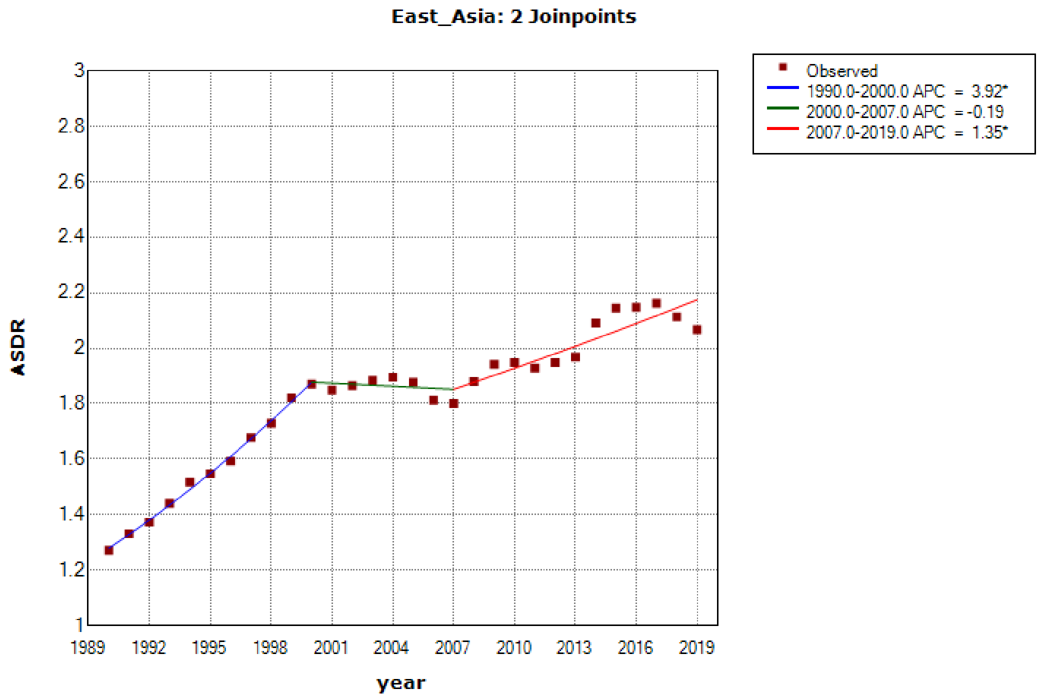

Figure S29. Trends in the age-standardized disability-adjusted life rate (ASDR, per 100,000 population) of PM2.5-related neonatal infections in East Asia from 1990 to 2019. *P<0.05.

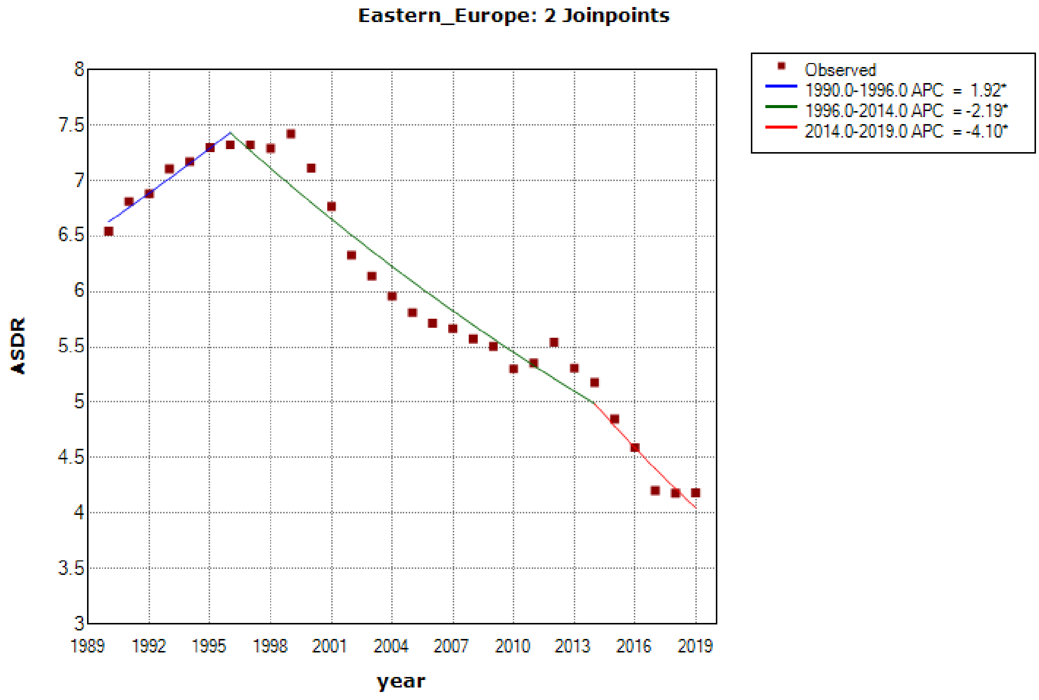

Figure S30. Trends in the age-standardized disability-adjusted life rate (ASDR, per 100,000 population) of PM2.5-related neonatal infections in Eastern Europe from 1990 to 2019. *P<0.05.

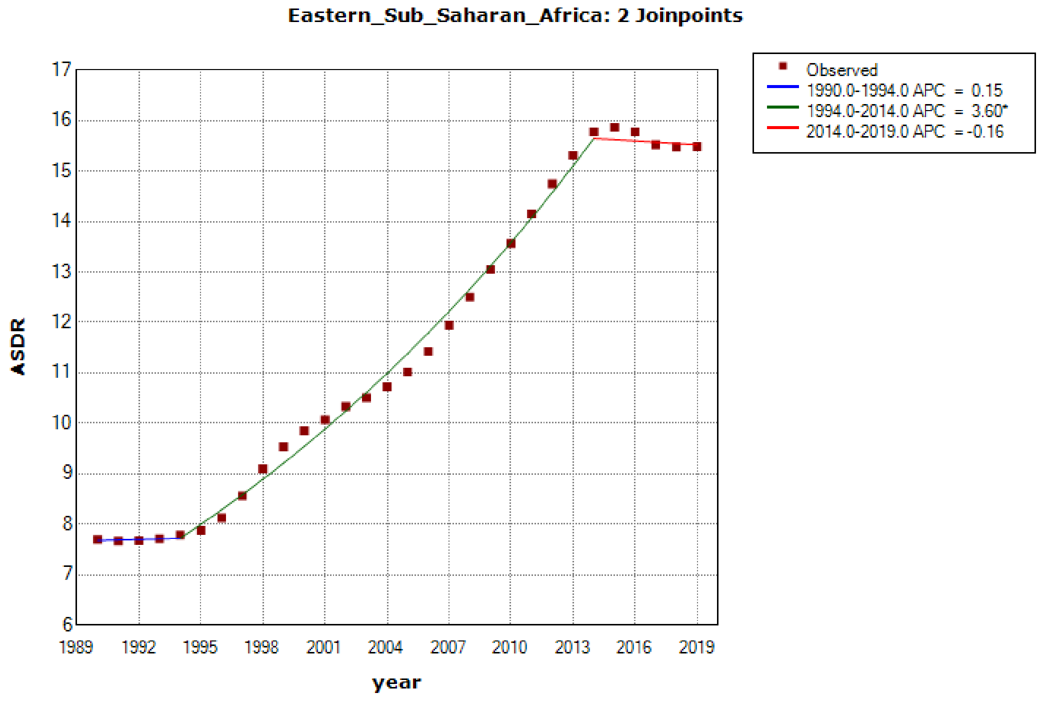

Figure S31. Trends in the age-standardized disability-adjusted life rate (ASDR, per 100,000 population) of PM2.5-related neonatal infections in Eastern Sub Saharan Africa from 1990 to 2019. *P<0.05.

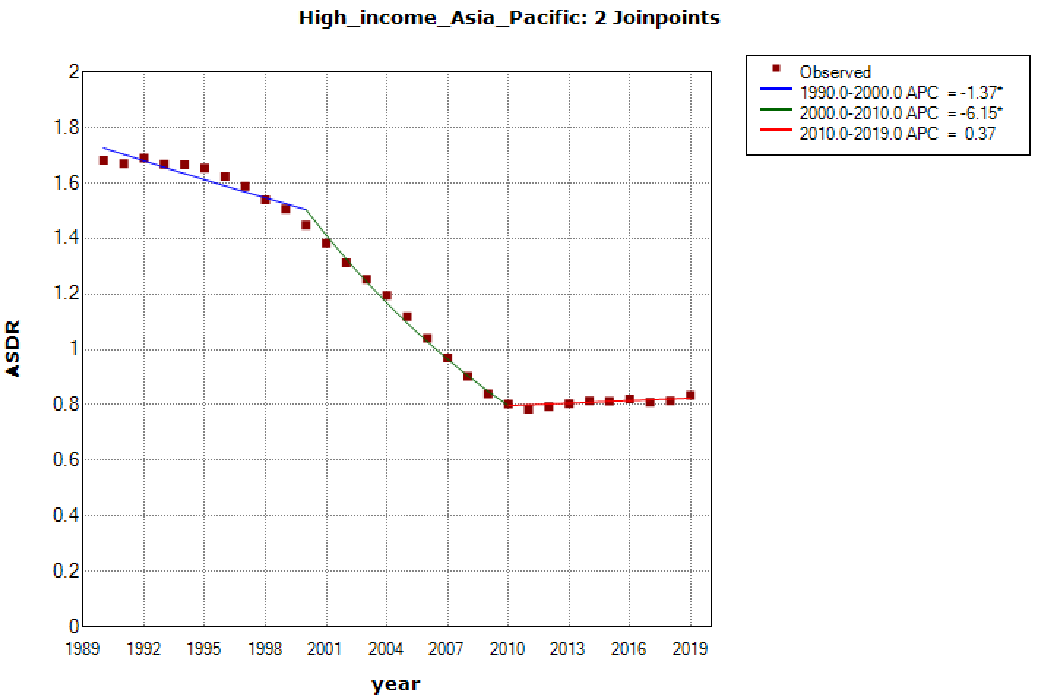

Figure S32. Trends in the age-standardized disability-adjusted life rate (ASDR, per 100,000 population) of PM2.5-related neonatal infections in High income Asia Pacific from 1990 to 2019. *P<0.05.

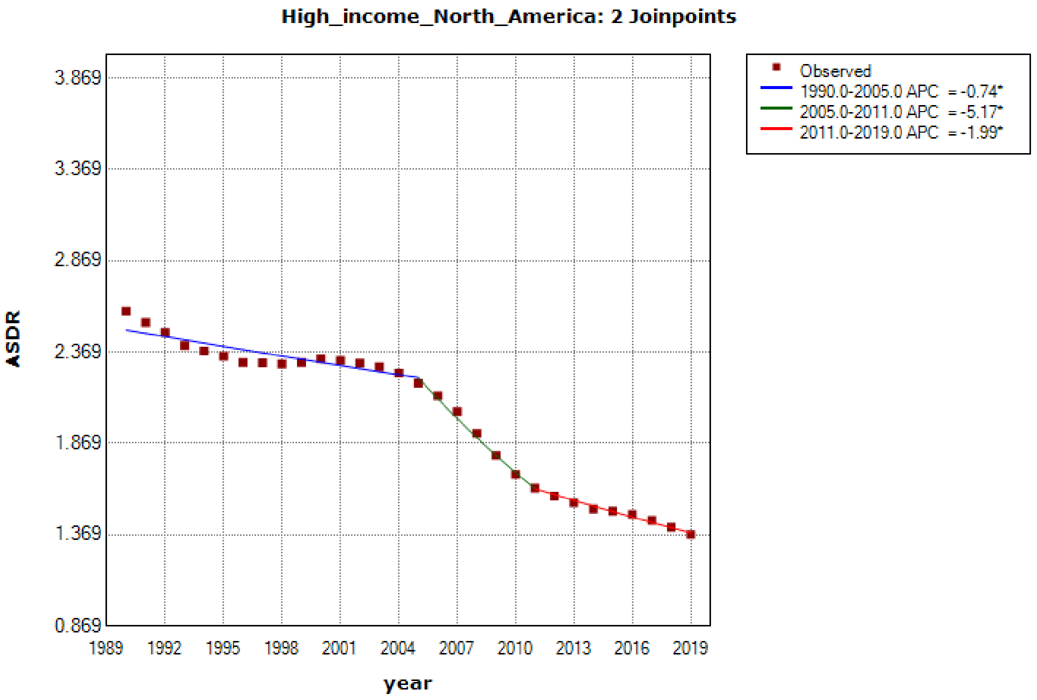

Figure S33. Trends in the age-standardized disability-adjusted life rate (ASDR, per 100,000 population) of PM2.5-related neonatal infections in High income North America from 1990 to 2019. *P<0.05.

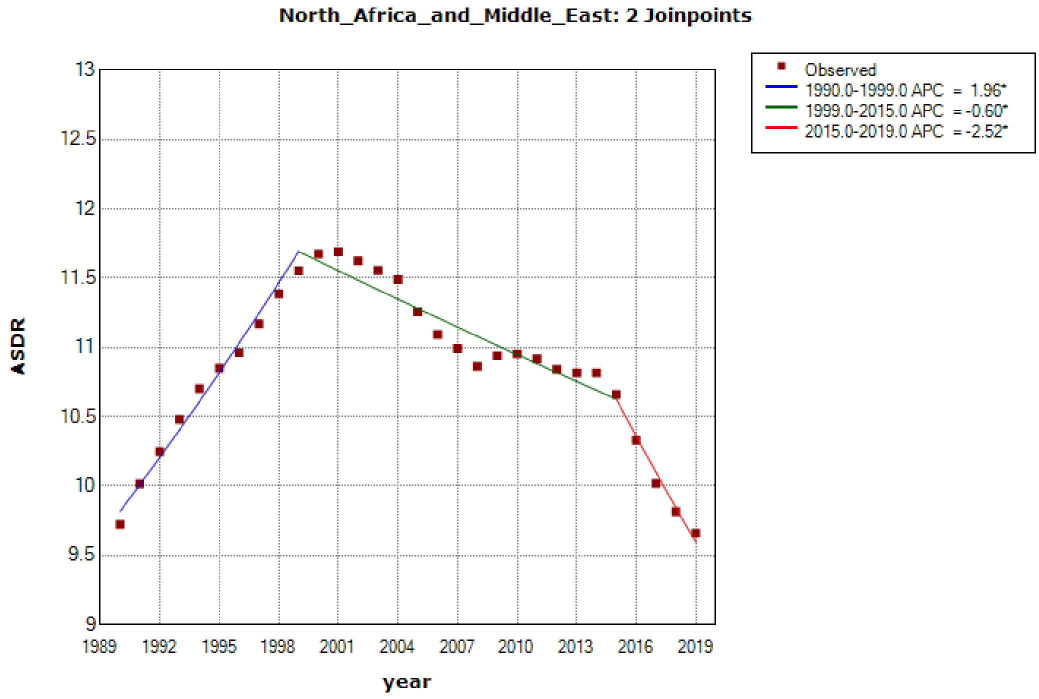

Figure S34. Trends in the age-standardized disability-adjusted life rate (ASDR, per 100,000 population) of PM2.5-related neonatal infections in North Africa and Middle East from 1990 to 2019. $^{*}P$<0.05.

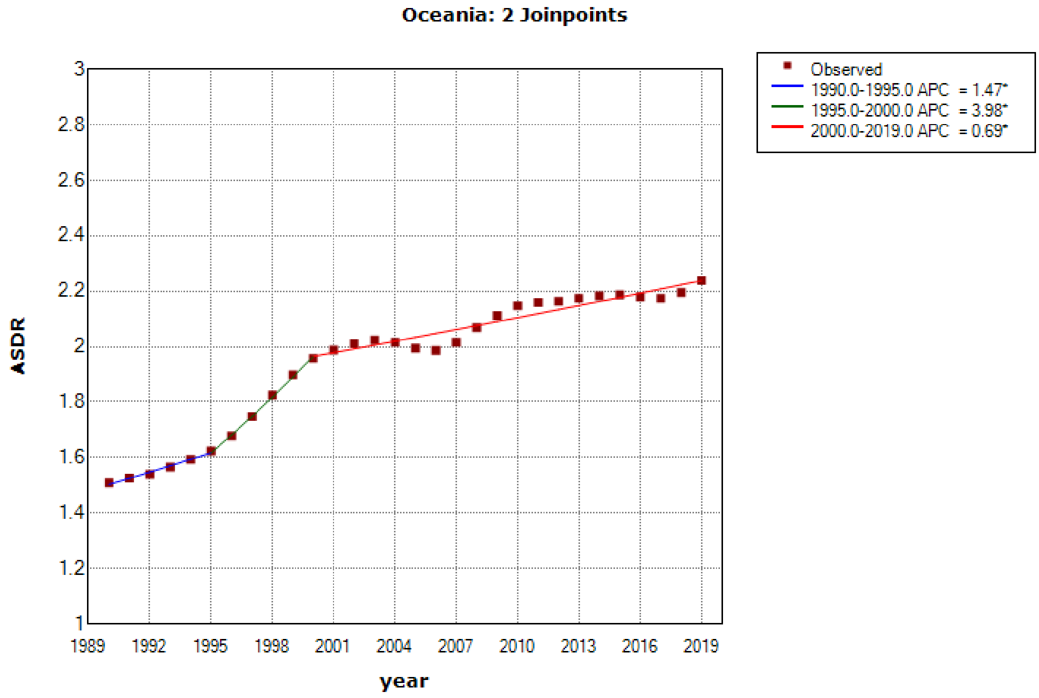

Figure S35. Trends in the age-standardized disability-adjusted life rate (ASDR, per 100,000 population) of PM2.5-related neonatal infections in Oceania from 1990 to 2019. $^{*}P$<0.05.

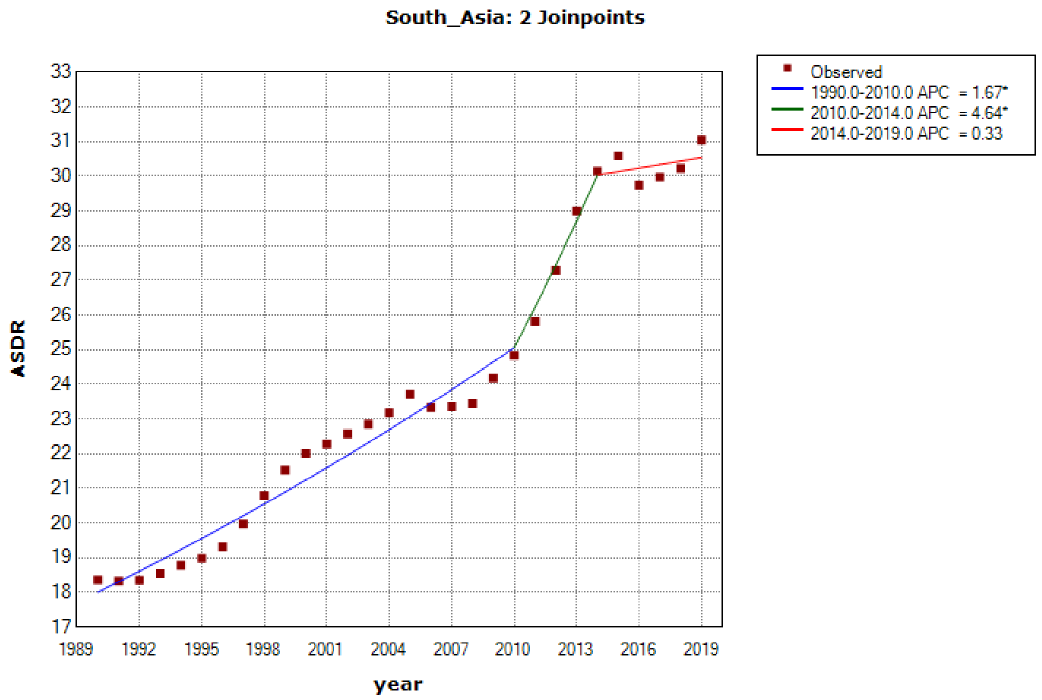

Figure S36. Trends in the age-standardized disability-adjusted life rate (ASDR, per 100,000 population) of PM2.5-related neonatal infections in South Asia from 1990 to 2019. $^{*}P$<0.05.

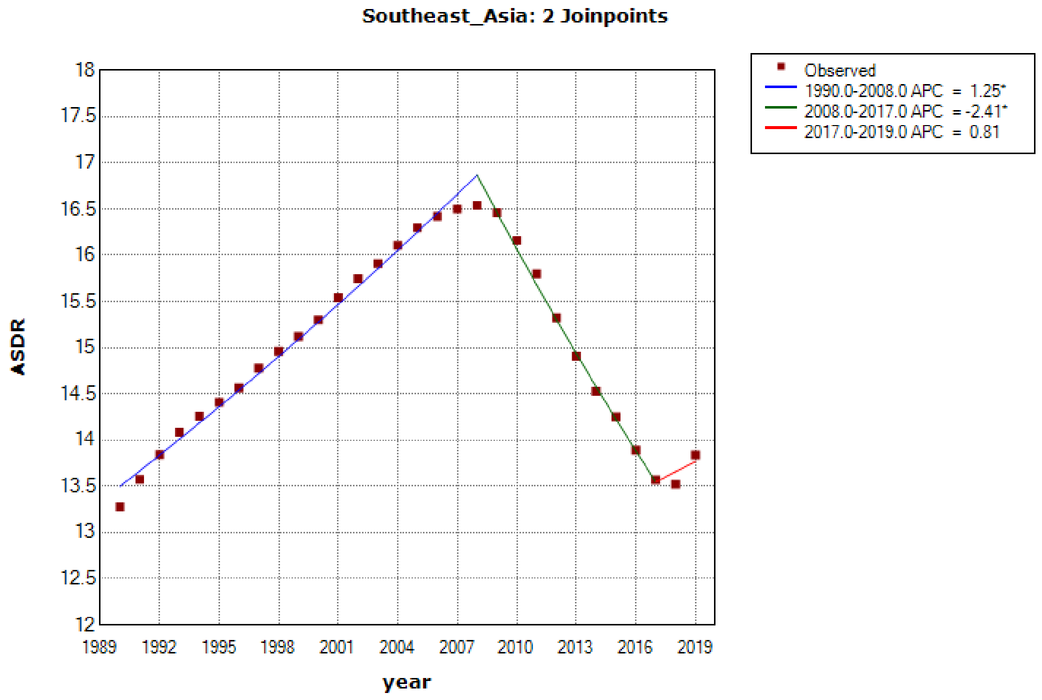

Figure S37. Trends in the age-standardized disability-adjusted life rate (ASDR, per 100,000 population) of PM2.5-related neonatal infections in Southeast Asia from 1990 to 2019. $^{*}P$<0.05.

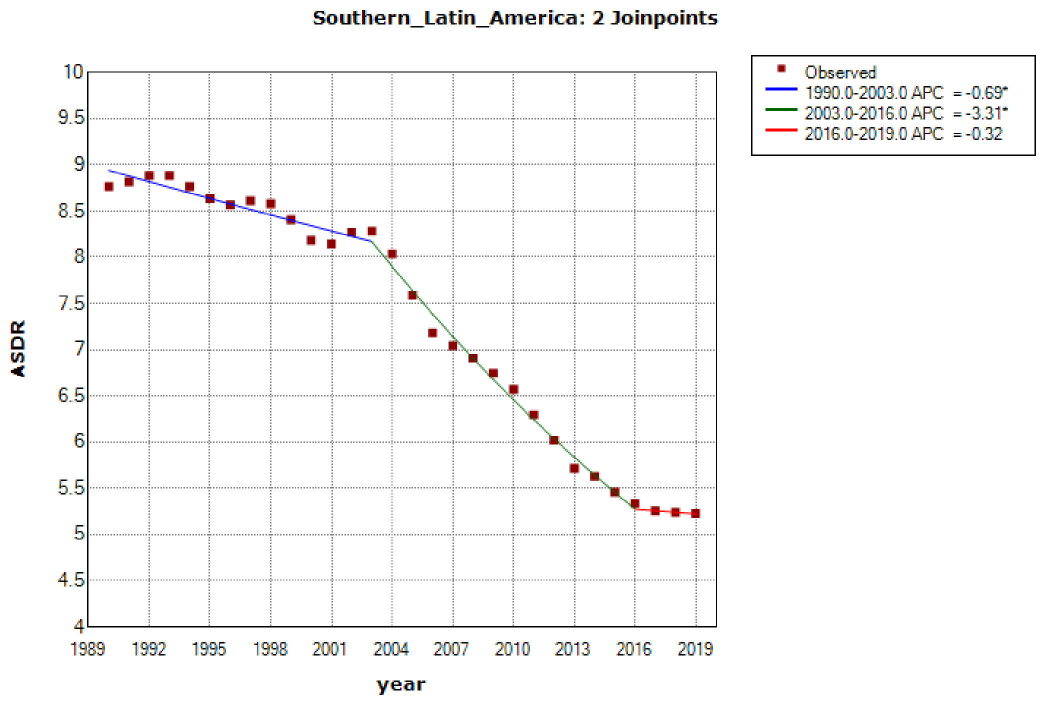

Figure S38. Trends in the age-standardized disability-adjusted life rate (ASDR, per 100,000 population) of PM2.5-related neonatal infections in Southern Latin America from 1990 to 2019. $^{*}P$<0.05.

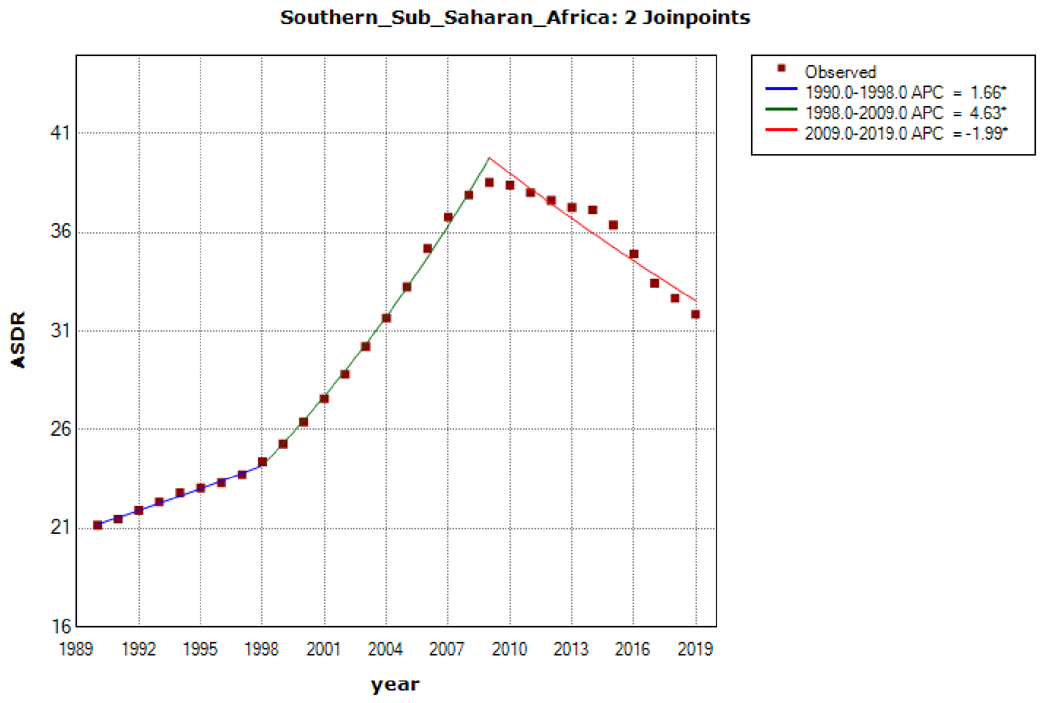

Figure S39. Trends in the age-standardized disability-adjusted life rate (ASDR, per 100,000 population) of PM2.5-related neonatal infections in Southern Sub Saharan Africa from 1990 to 2019. $^{*}P$<0.05.

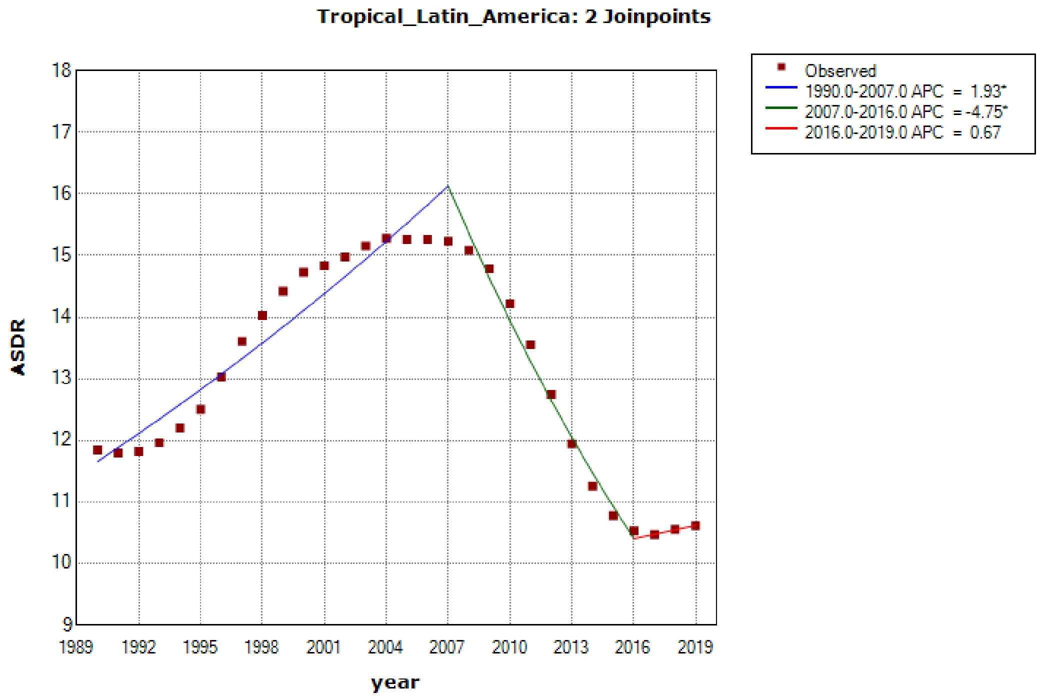

Figure S40. Trends in the age-standardized disability-adjusted life rate (ASDR, per 100,000 population) of PM2.5-related neonatal infections in Tropical Latin America from 1990 to 2019. $^{*}P$<0.05.

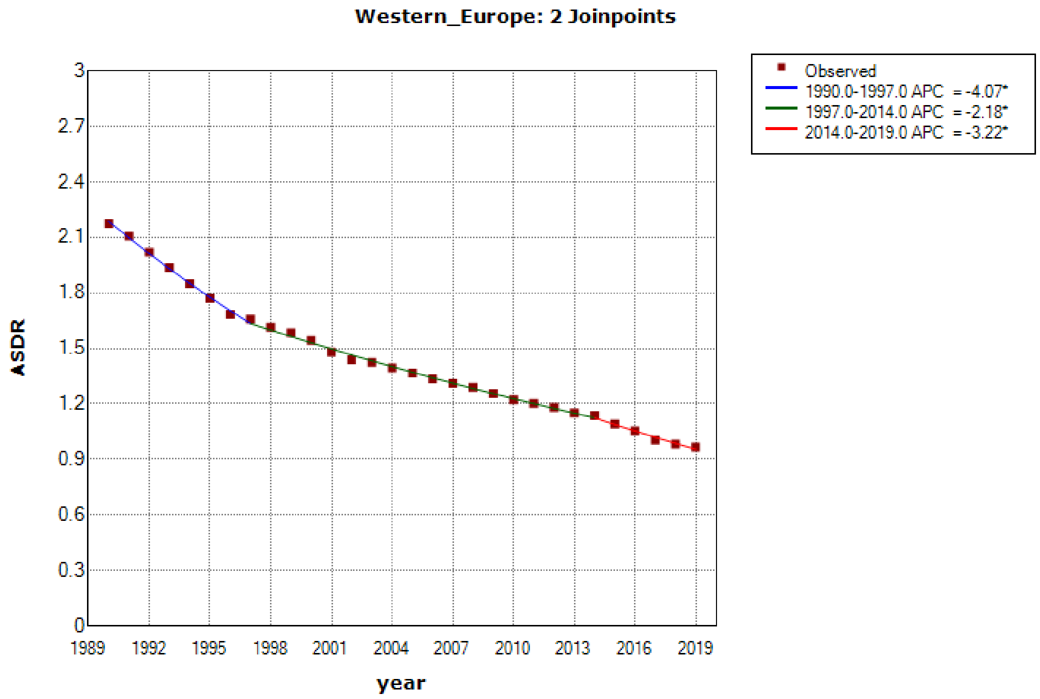

Figure S41. Trends in the age-standardized disability-adjusted life rate (ASDR, per 100,000 population) of PM2.5-related neonatal infections in Western Europe from 1990 to 2019. $^{*}P$<0.05.

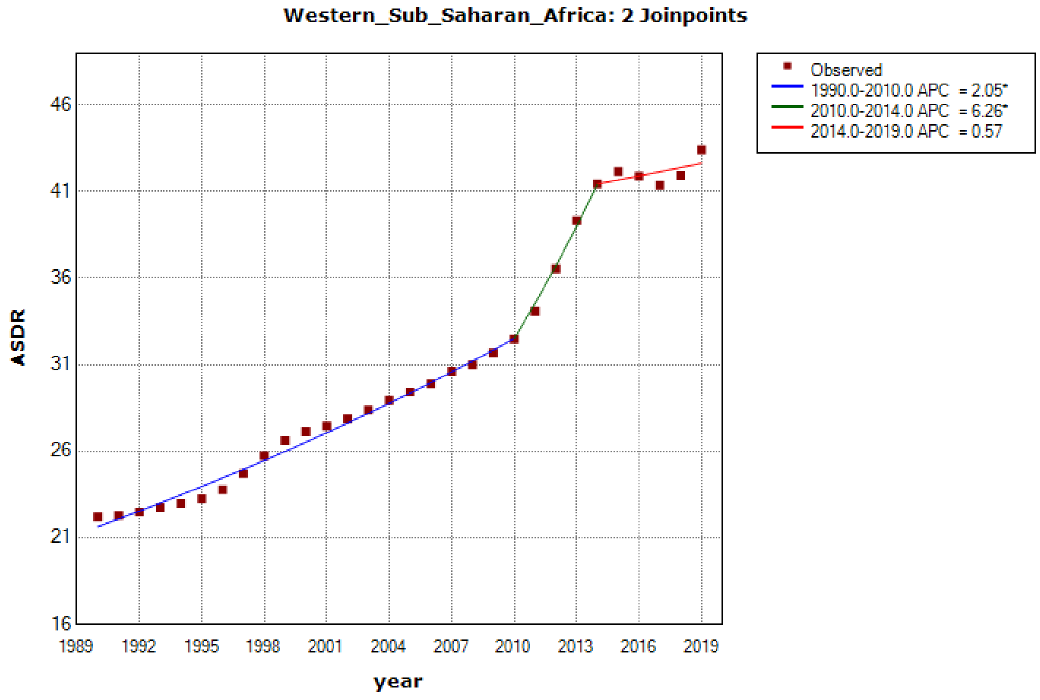

Figure S42. Trends in the age-standardized disability-adjusted life rate (ASDR, per 100,000 population) of PM2.5-related neonatal infections in Western Sub Saharan Africa from 1990 to 2019. $^{*}P$<0.05.

Part 3 (Figures S43–S45)

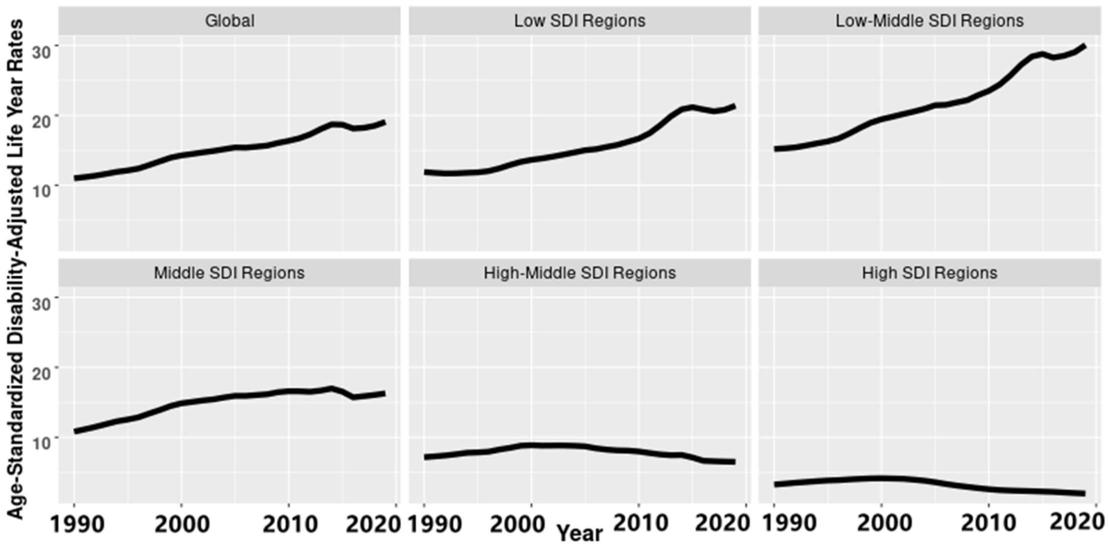

Figure S43. The changes of age-standardized disability-adjusted life years rate in PM2.5-related neonatal infections in the global and various socio-demographic index (SDI) regions from 1990 to 2019.

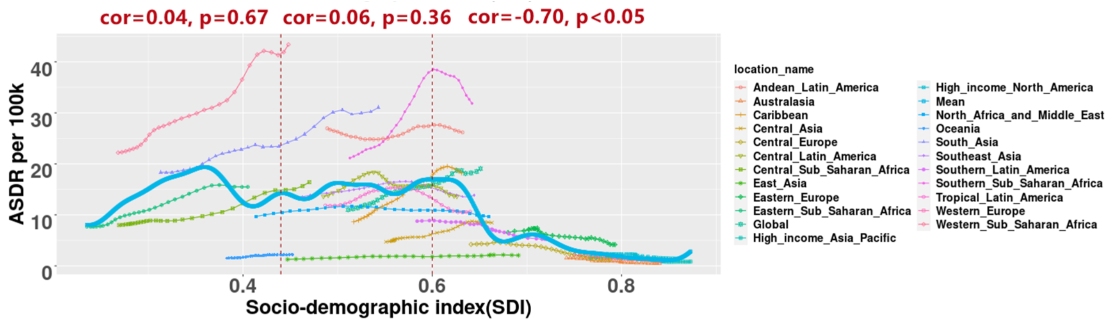

Figure S44. Age-standardized disability-adjusted life years rates (ASDR) of PM2.5-related neonatal infections in the global and 21 regions in 1990-2019. The expected value is represented by the solid blue line. The trends between ASDR and the socio-demographic index are represented by the Pearson correlation coefficient and p-value in the picture. The ASDR of PM2.5-related neonatal infections in four regions (South Asia, Southern Sub-Saharan Africa, Western Sub-Saharan Africa, Andean Latin America) were obviously higher than the expected value.

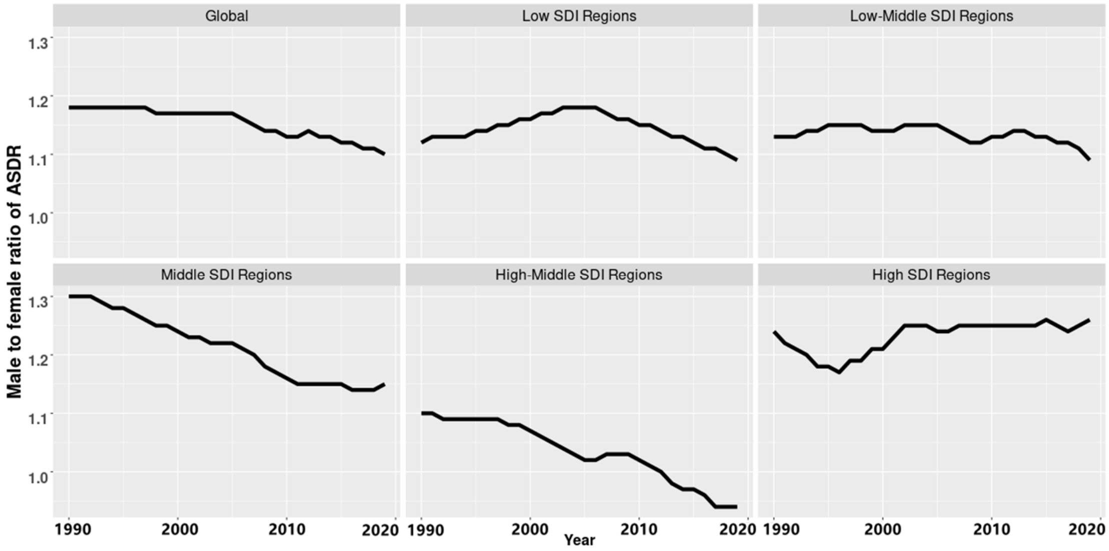

Figure S45. The ratio of male to female in age-standardized disability-adjusted life years rate (ASDR) for PM2.5-related neonatal infections in different socio-demographic index(SDI) regions in 1990-2019.
